# Nanofood Process Technology: Insights on How Sustainability Informs Process Design

**DOI:** 10.1021/acssuschemeng.3c01223

**Published:** 2023-07-24

**Authors:** Volker Hessel, Marc Escribà-Gelonch, Svenja Schmidt, Nam Nghiep Tran, Kenneth Davey, Lina A. Al-Ani, Nurhidayatullaili Muhd Julkapli, Yasmin Abdul Wahab, Ibrahim Khalil, Meng Wai Woo, Sally Gras

**Affiliations:** † School of Chemical Engineering, The University of Adelaide, Adelaide 5005, SA, Australia; ‡ EPS−School of Chemical Engineering, University of Lleida, Igualada 08700, Spain; § Nanotechnology and Catalysis Research Centre (NANOCAT), Institute for Advanced Studies, University Malaya, Kuala Lumpur 50603, Malaysia; ∥ Healthcare Pharmaceuticals Limited, Rajendrapur, Gazipur 1741, Bangladesh; ⊥ Department of Chemical & Materials Engineering, University of Auckland, Auckland 1142, New Zealand; # Department of Chemical Engineering and Bio21 Molecular Science and Biotechnology Institute, University of Melbourne, Melbourne 3010, Australia

**Keywords:** food processing, nanofood technology, nanostructures, nanoparticles, sustainability, sustainability assessment

## Abstract

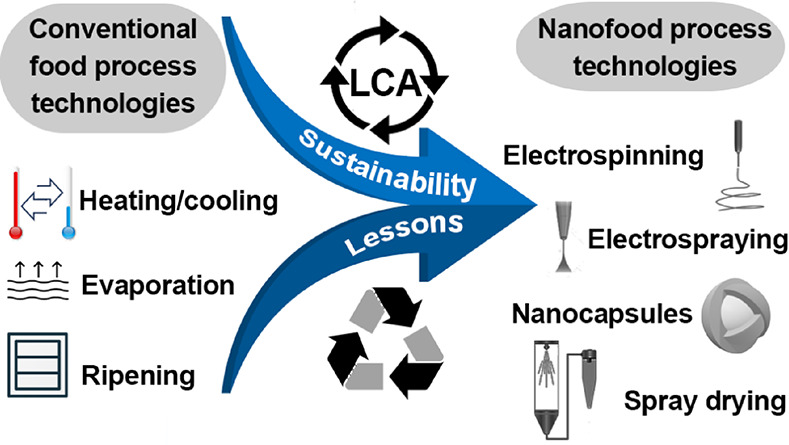

Nanostructured products are an actively growing area for food research, but there is little information on the sustainability of processes used to make these products. In this Review, we advocate for selection of sustainable process technologies during initial stages of laboratory-scale developments of nanofoods. We show that selection is assisted by predictive sustainability assessment(s) based on conventional technologies, including exploratory *ex ante* and “anticipatory” life-cycle assessment. We demonstrate that sustainability assessments for conventional food process technologies can be leveraged to design nanofood process concepts and technologies. We critically review emerging nanostructured food products including encapsulated bioactive molecules and processes used to structure these foods at laboratory, pilot, and industrial scales. We apply a rational method *via* learning lessons from sustainability of unit operations in conventional food processing and critically apportioned lessons between emerging and conventional approaches. We conclude that this method provides a quantitative means to incorporate sustainability during process design for nanostructured foods. Findings will be of interest and benefit to a range of food researchers, engineers, and manufacturers of process equipment.

## Abstract 2

Sustainability learning from conventional food process technologies informs nanofood process technologies.

## 1 Introduction

Food processing is evolving in its transformation of agricultural raw materials, preservation of fresh and perishable foods, and development of global trade. With the emergence of new technologies, an increasing variety of foods is being manufactured including instant soups and extruded cereals and foods with increased shelf stability and health benefit(s). Importantly, food processing is a matter of “social discussion”, with “ultra-processed foods” correlating with the prevalence of obesity^1^ and an increasing awareness of the need for sustainability.

The production of nanofoods uses operations common to nanotechnology in other industries, as well as operations from the food industry including the selection of raw materials, design of product(s) for new markets, structural analyses, and promotion of safety.^2−5^ Such emerging technologies, however, face the challenge of the need to replace or supplement well-known and well-defined conventional technologies. A driver for their acceptance is the design new food process technologies with enhanced sustainability.

Sustainability in this context is defined as “process methods and techniques, together with policies that support safety, economic and environmental objectives without placing future potential resources at risk to meet and customize individual human requirements in a way circular and environmentally friendly”.

It is hypothesized that knowledge and understanding, i.e., learning lessons, can be drawn from the findings of a significant number of existing sustainability studies of conventional food technologies. This learning is done not by direct transfer of knowledge but rather *via* joint unit operations,^6^ meaning the breaking down of the sustainability lessons to a particular level. This can be done in combination with predictive sustainability tools used in exploratory *ex ante*, “anticipatory” life cycle assessment(s).^7^

Food processing uses unit operations to provide sequential specific changes to raw materials to achieve desired internal properties and deliver palatable and highly nutritional foods demanded in part *via* consumers and market trends. Understanding this requires knowledge of mass, energy, and momentum transfer, together with the material properties of process intermediates. Food operations can be synergistically combined in a methodology called “hurdle” technology. The name stems from the accumulation of effects to set a hurdle to prevent negative effects on food, e.g., prevent deterioration from bacteria and increase food preservation.

A major goal of nanofood process technology is the dispersion of phases into each other to produce a structured, multi-phase system. Food materials, dispersions, and food processing media have three (3) major physical states, namely, gas, liquid, and solid. An additional fourth state of matter, plasma,^8^ has also only recently been reported in food processing as a medium.^9^ Another goal of nanofood process technology is to provide the targeted delivery of valuable compounds in a manner similar to what the pharmaceutical industry does with medicine(s). Various unprocessed foods contain nutraceuticals, i.e., bioactive compounds, that provide beneficial components or that positively impact overall well-being.^10^ Clinical studies have shown a correlation between a daily intake of such nutraceuticals and increased health.^11^ Examples include polyunsaturated lipids, vitamins, phytosterols, curcuminoids, carotenoids, and flavonoids.^9^ However, nutraceuticals are typically found at low concentration in foods derived from plants and animals.^12^ Delivery may also be compromised by chemical instability, low solubility in water, low bioavailability, and/or tightly bound compounds in a food matrix.^11b,13^ Nutraceuticals can also be altered by poorly controlled food processes and also during digestion.^14^ The food industry and nutritionists have developed “functional foods”,^10^ aiming to increase the concentration of nutraceuticals in foods or to add nutraceuticals to foods, i.e., to fortify foods.^15^ The processing and handling of nutraceuticals are not simple, however, as adding nutraceuticals directly to a food does not predicate desired results.^13^ Encapsulation of nutraceuticals prior to incorporation in a fortified food can give physical and chemical stability and control release at the desired site of action.^11b^ A current drawback of these processing methods for dispersion of phases and delivery of nutraceuticals is that there is little information on the sustainability of nanofood process technology.

In this Review, we (1) appraise the use of food materials, lipids, and biopolymers for forming specific nanostructures including emulsions, liposomes, encapsulates and hydrogels, and nano-architectures and (2) critically assess the transfer of concepts to industrial nanofood technology. For selected nanofood process cases, we (3) determine and quantify the sustainability of joint unit operations with conventional technology and (4) advocate for the selection of sustainable process technologies during initial laboratory-scale developments. We (5) show that selection is assisted by predictive sustainability assessment(s) based on conventional technologies, including exploratory *ex ante* and “anticipatory” life-cycle assessment, and (6) demonstrate that sustainability assessments for conventional food process technologies can be leveraged to design nanofood process concepts and technologies. We (7) critically review nanostructured foods at laboratory, pilot, and industrial scales, (8) apply a practical method *via* learning from the sustainability of conventional unit operations, and (9) apportion lessons between emerging and conventional approaches. We (10) conclude that this approach provides a means to incorporate sustainability during design for nanostructured foods and that the difficulty to developing a more generalized and quantitative methodology is that sustainability is unit operation- and food product-specific.

Findings will be of practical interest to a range of food researchers, engineers, and manufacturers of process equipment.

## 2 Nano-Architectures for Food Preparation

Nanofoods are developed from a wide range of ingredients including lipids, polysaccharides, and proteins. These are used to form a range of materials with varying properties including emulsions, liposomes, and particles.

The range of ingredients, materials, and properties highlight the conceptual diversity of formed nanostructures spanning from nanoemulsions to nanoliposomes. Most findings of nanofoods to date are broadly descriptive. These are explored for a range of food nanostructures below.

### 2.1 Nanoemulsions and Nanoliposomes from Lipids

Lipid-based nanostructures were developed in the 1990s to address the limitations of lipid-based macrostructures including liposomes and emulsions.^16^

Lipid is a collective term for a group of natural molecules that are either insoluble or difficult to solubilize in water, including mono-, di-, and triglycerides, fats, waxes, sterols, and phospholipids.^12,17^ Using a lipid as a carrier material for a nanostructure has a number of advantages, including the natural occurrence of lipids in some foods, the essential requirements for lipids in the human diet, and the potential of lipids to provide stored energy.^12,17,18^ Lipids are acknowledged as safe as a food additive^12^ while potentially providing additional nutritional value as a natural ingredient.^18^ Because of their hydrophobic nature, they dissolve lipophilic bioactive compounds readily^18^ and lipid-based nanostructures can also encapsulate hydrophilic molecules. Fortified lipid-based nanostructures need to be stabilized with food-grade surfactants, in which selection depends on the nanostructure, lipid, and food. Ionic surfactants and biopolymers are generally not suited to food-grade applications because of the toxicity of surfactants and the tendency to induce gelation of biopolymers.^12,19^ Food-grade options therefore include the use of non-ionic synthetic surfactants, with known exceptions, e.g., few natural proteins, such as latherin, are also suited.^12^

#### 2.1.1 Nanoemulsions and Microemulsions

**Figure 1:**
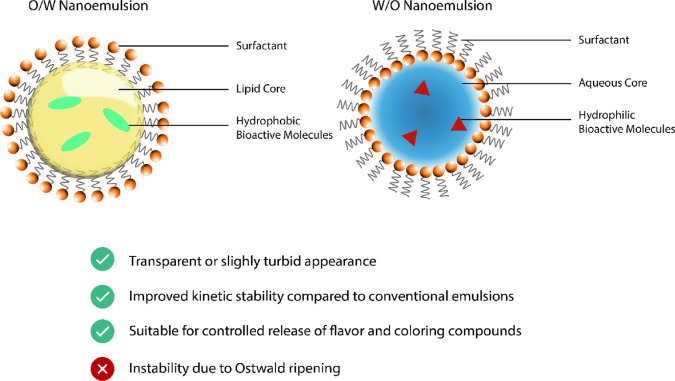
Oil-in-water (O/W) nanoemulsion and water-in-oil (W/O) nanoemulsion encapsulating hydrophobic bioactive molecules in a lipid core and hydrophilic bioactive molecules in an aqueous core, respectively.

The structure of nanoemulsions consists of two (2) immiscible (polar/non-polar) phases, with one dispersed in the other in which resulting droplets are stabilized *via* an emulsifier that is similar to a conventional emulsion. Made of oil and water, these structures form either oil-in-water (O/W) nanoemulsions common in food applications or water-in-oil (W/O) nanoemulsions that are more valuable in cosmetic application(s). In OW, oil is the dispersed phase and water is the continuous phase, while in WO, water is the dispersed phase and oil is the continuous phase (Figure 1). These structures allow bioactive molecules to be carried in the lipid, or aqueous, phase. Compared with conventional emulsions, the characteristic diameter of droplets in a nanoemulsion is typically 20 to 200 nm.^10,11^ Because of the “small” size of the droplets, there is no (or not apparent) scattering of light, giving nanoemulsions a near transparent appearance.^19a,20,21^ Nanoemulsions are therefore useful for optically clear food products including fortified soft drinks and bottled water.^21^

Nanoemulsions, as all emulsions, are not fully stable, meaning in thermodynamic equilibrium, because of coalescence and Ostwald ripening. Droplets get larger with time, adjusting the chemical potential of the droplets to maximize Gibbs free energy. The smaller the droplets, the more significant it is to this effect, and consequently, Ostwald ripening has to be reduced as much as possible to increase the long-term shelf stability of a nanoemulsion.^22^

Nanoemulsions have several practically useful functional properties. They are reported to boost the bioavailability of lipophilic bioactive compounds, i.e., the absorption in the gastrointestinal tract and uptake by cells, allowing controlled release at the intended site of action.^21,23^ Nanoemulsions are reported to control the release of flavor and coloring compounds that are susceptible to oxidative and photolytic degradation because of aldehyde, ketone, and ester groups.^24^ For example, food coatings based on nanoemulsions are reported to improve visual appearance and aroma perception, together with extending shelf-life by incorporating antioxidants, enzymes, antimicrobials, and antibrowning agents.^24^

**Figure 2:**
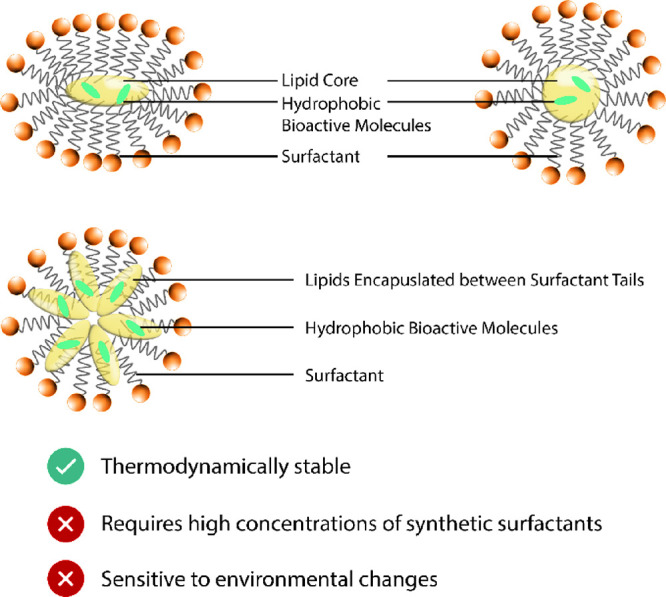
Oil-in-water (O/W) microemulsions encapsulating hydrophobic bioactive molecules in a lipid core or between surfactant tails.

Microemulsions contain droplets of a similar size of 2 to 100 nm.^21^ The name can be confusing, because the droplets are not in the micrometer range and are less than those for nanoemulsions. Microemulsions are different to nanoemulsions, because while these consist of the same compounds, oil phase, aqueous phase, surfactant, and often co-surfactant,^11b,25^ microemulsions are thermodynamically stable, i.e., the phases do not separate over time, and assembly is kinetically driven.^11b^ The droplets in a microemulsion can be spherical and non-spherical, and lipids encapsulated either as a core or between tails of surfactants (Figure 2).^25^ However, the state of the microemulsion, i.e., the Windsor Types, is highly dependent on environmental conditions and concentration ratio(s); the system therefore can undergo unwanted changes because of changing temperature or dilution.^11b,21^ Microemulsions need comparatively high concentrations of synthetic surfactant;^24^ this therefore limits application(s) in the food industry.^19a^

#### 2.1.2 Nanoliposomes

**Figure 3:**
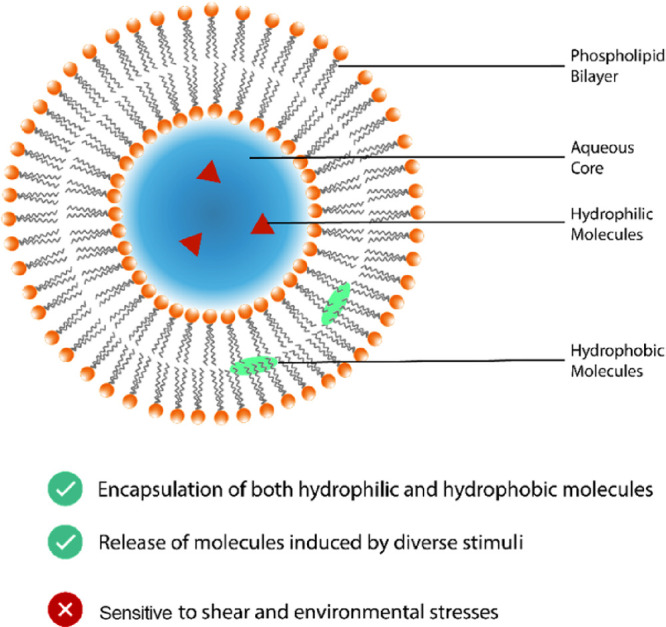
Nanoliposome encapsulating both hydrophilic and hydrophobic bioactive molecules in an aqueous core and phospholipid bilayer.

Nanoliposomes are spherical vesicles made from an aqueous core and enclosed by one or more lipid bilayers^26^ constructed from a lipid-based surfactant, such as a phospholipid that assembles when mixed with water under conditions of low shear stress.^26b^ This structure allows both hydrophilic and lipophilic compounds to be encapsulated,^27^ respectively, within the aqueous core or hydrophobic phospholipid tail (Figure 3). Select nanoliposome structures are reported to be biocompatible and biodegradable.^27^

Nanoliposomes are widely reported for pharmaceutical application. These are practically promising structures for use in foods, although a reported susceptibility to shear means some limitations. They offer practical promise, however, for the delivery of biomolecules,^28^ including dermal applications, because of the apparent ease with which nanoliposomes penetrate skin^26a,29^ and the range of stimuli that can be used to trigger delivery,^30^ including pH, enzymes, glucose, hyperthermia, ultrasound and light, or application of a magnetic field.^27b^ Nanoliposomes potentially encapsulate both hydrophilic and lipophilic nutraceutical ingredients, flavoring agents, enzymes, and microorganisms.^27a^ For widespread application, however, the stability of these under conditions of shear and environmental stress needs to be increased, because these can lead to the leakage of bioactive loads.^26^

#### 2.1.3 Solid-Lipid Nanoparticles

**Figure 4:**
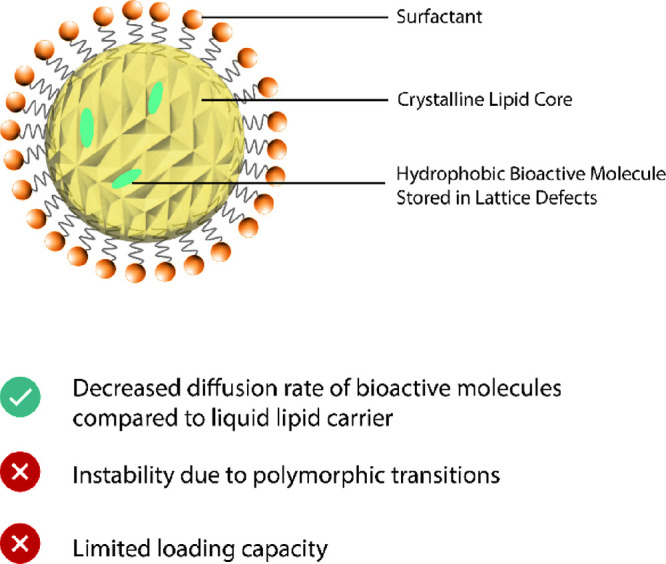
Solid-lipid nanocarrier (SLN) encapsulating hydrophobic bioactive molecules in lattice defects of a crystalline lipid core.

Solid-lipid nanoparticles (SLNs) are spherical particles in which the lipid carrier is solid at ambient and body temperatures.^18,26b,31^ In this way, the lipid carrier retains a crystalline structure following ingestion.^18^ The crystalline structure is important to encapsulation of bioactive molecules, namely, these are initially embedded in the lipid carrier in the form of a fortified nanoemulsion that is formed at temperatures above the melting point.^11b,12^ The nanoemulsion is then cooled, leading to crystallization of the lipid carrier with bioactive molecules entrapped within the defects of the crystal lattice.^11b,12^ The diffusion rate for the bioactive molecules is significantly decreased compared with a liquid lipid carrier, protecting the bioactive molecules from untimely release, together with prolonged release at the intended site of action (Figure 4).^19a^

The choice and composition of the lipid carrier and resulting crystallization behavior influence loading capacity and “release behavior”, together with determining SLN parameters including emulsification temperature and cooling rate.^11b^ In general, lipids that exhibit polymorphism are preferred, e.g., triacylglycerides (TAGs), because these allow for a greater number of lattice defects, allowing a greater number of bioactive molecules to be included within the nanostructure.^11b,18^

A major disadvantage with SLNs is a decreased loading capacity compared with other nanostructured systems, because the crystalline structure limits the number of bioactive molecules that are incorporated.^11b,19a,26b^ Importantly, the polymorphic nature of the lipid carrier can be a practical difficulty because polymorphic transitions during storage can lead to drug expulsion, called “burst release”,^19a,32^ or induce gelation.^33^

#### 2.1.4 Nanostructured Lipid Carriers

**Figure 5:**
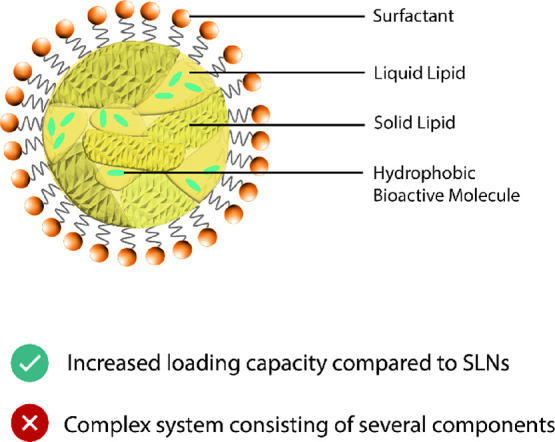
Nanostructured lipid carrier (imperfect type) encapsulating hydrophobic bioactive molecules in liquid lipid sections.

Nanostructured lipid carriers (NLCs) were developed to overcome limitations of SLNs, and they are described as modified SLNs.^26b^ The carrier matrix is not (exclusively) made of crystalline lipids but also consists of a mixture of lipids with melting points above and under a healthy body temperature (36.8 °C), leading to particles that contain both solid and liquid structures at room and body temperatures.^18^ To obtain an NLC lipid carrier matrix polymorph, solid lipids are mixed with unsaturated fatty acids at temperatures above the melting point.^18^ When the precursor nanoemulsion is cooled to induce solidification of the polymorph lipids, solidification is modified by the liquid components of the matrix. For example, for imperfect NLC with crystalline and liquid sections in the lipid core,^19a,26b^ the unsaturated fatty acids inhibit formation of highly ordered crystal lattices and increase the number of lattice defects compared with SLNs, increasing loading efficiency, which is the ratio of actual to theoretical bioactive nanomaterial content.^18^ Concurrently, many hydrophobic bioactives exhibit increased solubility in a liquid lipid compared to a solid lipid,^10^ easing incorporation of bioactives. However, the solid structures in NLC prevent diffusion of bioactives into the aqueous phase, i.e., prevent degradation from environmental influences, and allow a controlled release of bioactive at the intended site of action.^19a^ Selection and composition of the NLC lipid carrier matrix, together with the bioactive, significantly influence structural characteristics of the resulting nanostructure, e.g., crystalline packing and rigidness, and NLC production parameters.^18,19^ The liquid lipids are reported to “slow down” polymorphic transitions of the solid liquids, thereby improving stability.^19a,26b^ In particular cases, crystallization is inhibited, leading to an amorphous solid structure of solid lipid.^19a^ NLC is therefore a complex system, and components need to be selected carefully. Additionally, lipids like cholesterol or saturated lipids can be used as solid lipid in NLC, but this is undesirable, given the potential risk to health (Figure 5).^19a^

### 2.2 Nanohydrogels, Nanoencapsulates, and Nanoemulsions from Biopolymers

The term “biopolymers” describes polymers that are obtained from a natural source, e.g., animals or plants, and mostly refers to protein and polysaccharides. Biopolymers are preferred to synthetic polymers, many of which are not of food grade, because of association with inflammatory reactions and toxicity and are also costly to produce.^14a^ Most biopolymers that are obtained as by-product(s) or waste(s) in agriculture and the food industry are non-toxic. The use of biopolymers as a nanostructure matrix is different when using lipid-based nanostructures. While physicochemical characteristics of lipid-based nanostructures result from intermolecular interactions of its components, biopolymers are selected based on physicochemical properties of the polymer chains that are adjustable *via* intramolecular change(s). Therefore, biopolymer nanostructures and bioactive binding sites can be designed on a molecular level for selected application. While biopolymers exhibit practical promising characteristics for the design of nanostructures for the food industry, they are not often used.^11b,19a^ Practical difficulties include the low availability of biopolymers with consistent quality, required use of organic solvents during production, and the absence of large-scale production.^11b,34^ However, for functional properties, biopolymers are considered valuable as food additives and used as emulsifiers to stabilize lipid-based micro- and nanostructures. For example, Gum Arabic, a gum obtained from certain Acacia trees, is commonly used as an emulsifier in beverages.^34^

#### 2.2.1 Protein-Based Nanostructures

Proteins are biopolymers, with monomers of one or several amino acids. Animal and plant sources are commonly used in the food industry.^35^ Production can readily be incorporated into existing processes, giving opportunity to valorize former waste streams, e.g., producing gelatin from collagen found in the skin and bones of animals.^36^ Therefore, proteins are generally considered as inexpensive and label-friendly functional ingredients in processed foods while providing nutrition and being biodegradable, biocompatible, and non-toxic (GRAS, “Generally Recognized as Safe” by Food and Drug Administration).^14a,35^ Practical functionality includes emulsifying, jellifying, and foaming properties,^14a,37^ therefore significantly impacting sensory properties of final food product(s).^35^ These functional properties stem from molecular characteristics, i.e., the arrangement, number, and type of amino acid residues, the electrical charges in the biopolymer chain, and tertiary and quaternary structures that impact intra- and intermolecular bonding to influence hydrophobicity, aggregation, and network formation by the protein structure. Intermolecular bonding is also influenced by environmental conditions including temperature, pH, and ionic strength.^14a,38^

**Figure 6:**
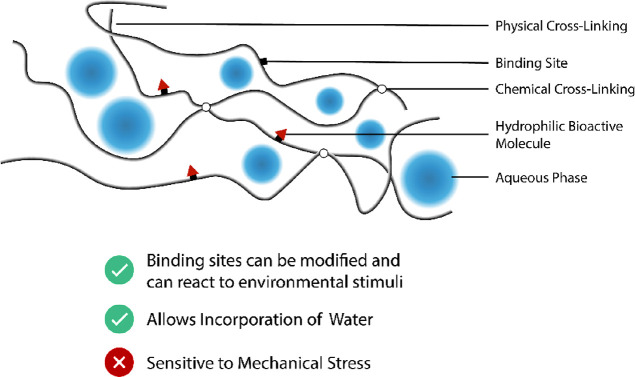
Swelled nanohydrogel with chemical and physical cross-linking and hydrophilic bioactive molecules at binding sites.

Specific protein nanostructures incorporated in a food can include hydrogels, solid particles, or emulsions.^35^ Nanohydrogels are especially of interest, as they can be formed readily from proteins without incorporating synthetic polymers. Hydrogels can also consist of polysaccharides or of a mixture of proteins and polysaccharides (Figure 6).^39^

#### 2.2.2 Polysaccharide-Based Nanostructures

**Figure 7:**
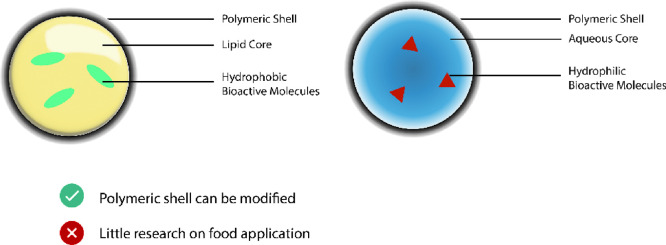
Examples of nanocapsules with a lipid or an aqueous core encapsulating hydrophobic bioactive molecules or hydrophilic bioactive molecules, among a variety of available techniques and materials.

Polysaccharides are a group of hydrophilic polymers in which monomers are monosaccharides connected *via* a glycosidic bond^13,40^ (Figure 7). Because of a natural origin from animals, plants, algae, and microorganisms,^41^ they share advantages with proteins including biocompatibility, biodegradability, renewability, availability, and (relatively) low toxicity.^13,41b,42^ In contrast with proteins, polysaccharides exhibit low immunogenicity.^41b^ As food additives, polysaccharides provide a range of functionalities including improving the thermostability of foods and modification of texture and structural properties.^43^ Polysaccharides are available in a myriad of forms, including hydrolyzed and functionalized forms, and are therefore widely used in the food industry. They are mainly sourced directly from agricultural ingredients. When sourced within the food industry from by-product streams, complex purification is sometimes needed because of complex polysaccharide structure(s) and other by-products and impurities.^41b^

Polysaccharide-based nanostructures for encapsulation have been more generally researched for controlled release of drugs in medical application(s). Consequently, many of the nanostructures intended for food fortification have originated from medical research and have been applied in the food industry. Because major groups of polysaccharides are not soluble in acids, polysaccharide-based nanostructures withstand the hazardous environment of the stomach and transport bioactive molecules to the colon, an important site in the gastrointestinal tract for absorption of bioactives. Among polysaccharide/biopolymer-based nanostructures are nanocapsules that are spherical vesicles with a polar, or a non-polar, core and a polymeric shell. For medical application, chitosan and alginate are used as shell materials. Biopolymer nanocapsules can also be obtained from proteins including zein and casein.^39^

Additionally, polysaccharides have been reported as potential functional additives in a range of inorganic nanostructures with matrix materials including, Au, Ag, C, and graphene, resulting in “polysaccharide-based nanocomposites”.^41b^

To better understand the selection of structures described in this section, Table 1 includes a summary of literature references of specific examples where each architecture is used, the construction materials, and the embedded nano-molecules applied to food processing.

**Table 1:** Examples of Different Architectures Made of Different Materials Used to Embed Valuable Nano-Molecules in Food Processing

architecture	main material	origin	embedded molecules	ref.
nanoliposomes from lipids	lecithin	egg yolk/soybean	vitamins C, D, and E	(44)
			curcumin	(45)
	phospholipids	milk	tea polyphenol	(46)
		soybean	essential oil	(47)
			vitamin D_3_	(48)
		egg yolk	carotenoids	(49)
	chitosan	sea food	essential oil	(50)
			vitamin C	(51)
			w-3 PUFAs (fish oil)	(52)
nanoemulsions	chitosan	sea food	curcumin	(53)
			trehalose	(54)
	gelatin	pork	carotenoid	(55)
			buriti oil	(56)
	lecithin	soybean	thymol	(57)
		chicken	rosemary extract, cinnamon essential oil	(58)
nanohydrogels	protein	whey	d-limonene	(59)
			caffeine	(60)
			iron	(61)
			folic acid	(62)
	β-lactoglobulin	milk	caffeine	(63)
			iron	(64)

## 3 Nanofood Process Technology and Sustainability

### 3.1 Sustainability Lessons from Conventional Food Technologies

The reported nanofood process technologies reviewed above lack adequate or sufficient information on potential process sustainability, as well as lacking life cycle assessments of the process steps used to structure and produce nanofoods. However, in this absence of understanding, useful comparisons are available from established sustainability studies of conventional food technologies that can be used for learning in nanofood technologies.

Both nanofood and conventional food processes share general dependencies on raw materials and regional resource supply and also share unit operations from chemical, mechanical, and electrical engineering. Usually, one of these operations determines process sustainability. Lessons can therefore be drawn between food processes at the level of this unit operation despite the potential differences in the details of technologies. An efficient heating concept, for example, will likely add efficiency when applied across different systems.

Table 2 presents the common engineering operations connected with nanofood processing technologies. It also includes a summary of the sustainability lessons that can be drawn from judicious consideration of shared engineering operations in nanofood technologies and conventional food processing. Sustainable aspects include energy, water, global warming potential, and terrestrial acidification, that is, quantification of changes in soil chemical properties caused by atmospheric emission of pollutants affected by processing; these include nitrogen oxides (NO_*x*_), ammonia (NH_3_), and sulfur dioxide (SO_2_). These gases impact ecosystem quality, leading to changes in pH that weakens plant growth and failure of seeds to germinate.^65^

**Table 2:** Summary of Sustainability Lessons Drawn from Engineering Operations Shared between Nanofood and Conventional Food Process Technologies

technology	engineering operation(s)	sustainability learned from conventional operation(s)
electrospinning	ohmic heating	reduction in energy demand
	electromagnetic activation	reduction of global warming potential and terrestrial acidification
	evaporation	wastewater reduction
		
electrospraying	ohmic heating	reduction in energy demand
	high pressure	
	electromagnetic activation	
		
spray drying	evaporation	reduction in energy demand
	heating	reduction of global warming potential and terrestrial acidification
	electromagnetic activation	wastewater reduction
		
desolvation	mechanical treatment	reduction in energy demand
		
nanocrystallization	heating	reduction in energy demand
	cooling	reduction of global warming potential and terrestrial acidification
	mechanical treatment	wastewater reduction
	electromagnetic activation	
	evaporation	
		
nanoemulsions	heating	reduction in energy demand
	cooling	reduction of global warming potential and terrestrial acidification
	mechanical treatment	wastewater reduction
	ultra-sound	
	evaporation	
		
nanoliposomes	mechanical treatment	reduction in energy demand
	ultra-sound	
	evaporation	
	supercritical CO_2_	
		
operations not used so far in nanofood processing	air/sun drying	
	osmotic pressure	
	microwaves	
	vacuum impregnation	
	plasma	

Nanofood processing therefore needs to “adopt, adapt, and optimize” sustainable engineering operations. There are a number of lessons from conventional food process technologies that have been understood and have been or could be applied practically to nanofood development. Sustainability lessons are generally available *via* the impact of a main unit operation.

### 3.2 Generalized Sustainability Lessons

Important sustainability lessons for the food industry common to both conventional and nanofood processing include (1) greenhouse gas release (GHG), with the food industry responsible for *ca*. one-third of global emissions,^66^ (2) freshwater consumption, with food processing accounting globally for the third highest water consumption and wastewater discharge^67^ where in the United States, this represents *ca*. 80% of the total consumption,^68^ (3) energy consumption, *ca.* 5% of the total industry,^69^ and (4) chemical pollution, including that from mineral fertilizers and pesticides.^70^

#### 3.2.1 Raw Materials

Raw material production, including crop cultivation and animal husbandry, generates the most significant environmental impact within food production, including land-use change, reduction in biodiversity, freshwater eutrophication (overfertilization), global warming (fermentation gas emissions), water shortages (over irrigation), ecotoxicity, and human toxicity (fertilizers and pesticides).^71^

Food process technology contributes *ca*. 10 to 20% of the total environmental impact from the food industry.^72^ This will likely be decreased *via* process optimization(s) in common, global food unit operations, including drying, heating, and freezing.

#### 3.2.2 Regional Variance

Because ecosystems, nationally and locally, rely on geographical climatic and soil variance and farming practices, there are significantly different sustainability outcomes from Life Cycle Assessments (LCA).^72^ While some aspects of food processing are common regardless of geographical region, others vary, including the energy-mix. For example, the LCA impact of food processing using German energy sources of 15% nuclear and 55% fossil was 4 to 5× greater than French energy sources of 76% nuclear and 6% fossil.^73^

#### 3.2.3 Benchmarking of Food Unit Operations

Global warming potential (GWP) is an acknowledged measure for benchmarking the potential impact of essential unit operations in food manufacturing. The GWPs for three (3) common operations, namely, drying, heating, and cooling, are summarized comparatively in Table 3 for a range of selected foods.

**Table 3:** Comparative Summary of Global Warming Potential (GWP) for Selected Food Technologies

food technology	operation	product	GWP (kg CO_2_-eq kg^–1^)	reference
drying	drum-drying	apples	2.67	(74)
	freeze-drying	strawberries	1.54	(75)
	spray-drying	apple pulp	0.80	(74)
	infrared-drying	apricots	0.71	(76)
heating	pasteurization	milk	0.42	(77)
		cream	0.43	(78)
		cheese	1.65	(79, 80)
	ultra-heat treatment	milk	0.21–0.59	(78, 81)
	inoculation + incubation	yogurt	0.49	(78)
	evaporation	milk powder	1.60	(82)
	smoking	Galician cheese	1.92	(83)
cooling	freezing	beans	0.70	(84)
		broccoli	2.64	(84)

It is seen in the table that the value for GWP ranges from *ca*. 0.42 to 2.67 kg CO_2_-eq kg^–1^. This impact is significant and evident when understanding that it is similar in value to the synthesis of NH_4_ through the Haber–Bosch process (HBP). (Incidentally, this consumes the greatest energy in the chemical process industry with 1.5 to 3.0 kg CO_2_-eq kg^–1^.) The range of values relates both to specific diversification in unit operations, for example, drum, freeze, spray, and infrared forms of drying, together with food type, for example, apples, strawberries, apple pulp, and apricots. A drawback however is that the reported literature does not permit a quantitative separation of both these effects.

#### 3.2.4 Impact of Additional Food Unit Operations

The data of Table 3, while practically useful, present a simplified view with focus on individual unit operations. For a more complete assessment, the whole process including any additional unit operations needs to be considered.

**Figure 8:**
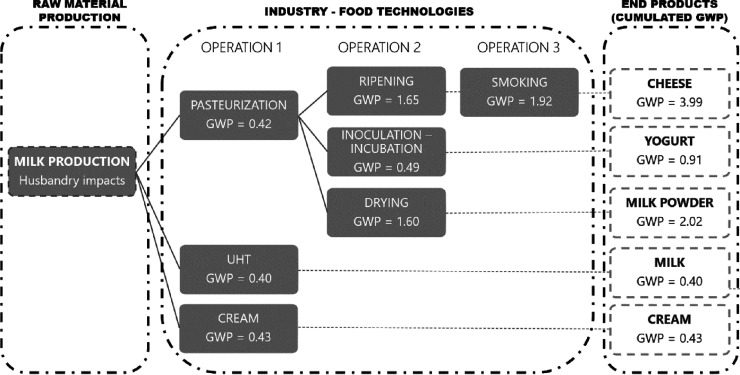
Schematic for accumulated GWP with successive unit operations in processing of milk to dairy products. This analysis demonstrates the effect of additional operations that sum to give the “total” impact in food manufacturing.

Because milk processing is widely studied and understood globally, it serves as a practically useful example. The cascade of unit operations used to produce a range of dairy products from milk is presented in Figure 8. A common environmental impact for all products arises from raw material production based on feeding of animals, solid wastes, and gaseous emissions of CH_4_. It can be usefully noted here that upstream operations, including animal or vegetable production, account for one-third of global greenhouse emissions (GWP)^66^ and 80% of freshwater consumption in the United States,^68^ while mineral fertilizers and pesticides are globally a main source of air and water pollution.^70^

In Figure 8, Operation 1 is a thermal treatment for milk preservation. GWP data are seen to be similar for different milk products, confirming that using the same important operation on different ingredients with varying protein, fat, and water content provides comparable sustainability for this operation. Once the milk is pasteurized, different operations can follow for milk-product diversification, unless milk and cream are the end-products. Operation 2 with ripening, inoculation/incubation, and drying increases GWP impact *ca*. 4× compared with Operation 1. The “smoking” product in Operation 3 has a significant GWP.

Maximal cumulated GWP for cheese is 10× greater than for milk when smoking is included (although this is not common for all cheeses), and yogurt is 2× that for milk, underscoring the significant impact of successive process steps.

## 4 Process-Specific Sustainability Lessons for Nanofood Processing

Operation-specific lessons for the sustainability of nanofood products are especially important and include electrically, thermally (heating and cooling), and dispersion-driven processes.

### 4.1 Electrically Driven Nanofood Technologies and Operation-Specific Sustainability

Lessons can be drawn for nanofood structure generation from comparison with conventional processes of electrospinning and electrospraying and from conventional evaporation or drying.

#### 4.1.1 Electrospinning

**Figure 9:**
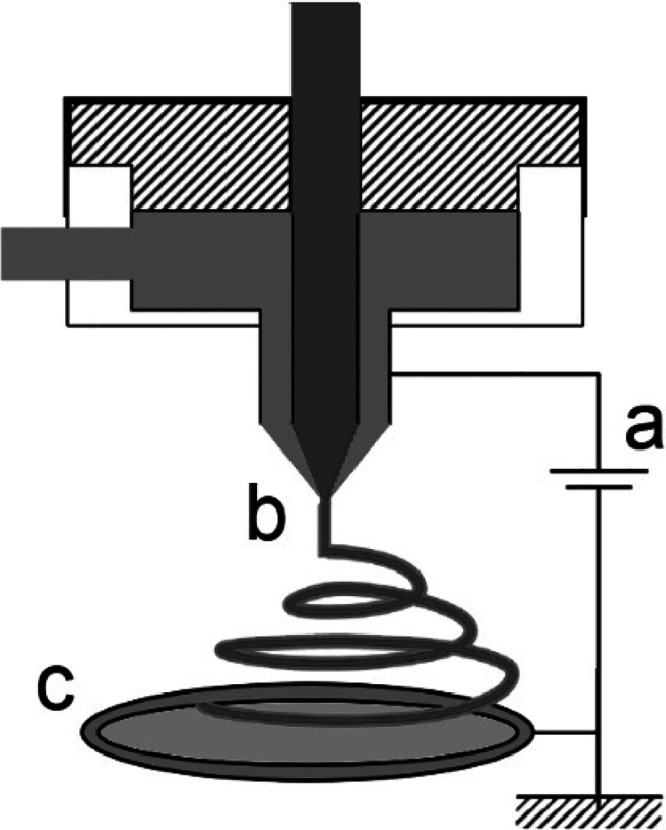
Coaxial spinning for fabrication of nanofibers. (a) High-voltage power supply, (b) coaxial jet, and (c) collector.

Electrospinning generates encapsulated solid nanoparticles when a fine powder particulate is dispersed in a spinning dope. Electrospuns are produced *via* injection of a polymer/biopolymer solution from a spinneret into a prepared collector,^85^ as shown in Figure 9. The nanofiber has a large surface-to-volume ratio and is used as a carrier for bioactive food ingredients and nutraceuticals.^86^ At the laboratory scale, a syringe pump is used to impulse the spinning liquid through a spinneret fabricated from a hypodermic needle. The needle is charged *via* electrical connection.^87^ Following the spinneret, a collector is placed that is (usually) a stationary metal plate or foil connected to the ground to remove residual surface charges.

Coaxial electrospinning is used to control the release of encapsulated food compounds including nutraceuticals, proteins and enzymes, or bacteria. For example, *Bifidobacterium* can be encapsulated in poly(vinyl alcohol) and used to produce 150 nm fibers.^88^ Two-component fibers are extruded from two (2) polymers using coaxial electrospinning with two spinnerets, one inside the other (Figure 9),^85,89^ where the inner core material has greater viscosity.^90^

A range of food-grade polymers is used for electrospinning to entrap, coat, or encapsulate proteins (e.g., casein, soy and whey, gelatin, albumin, collagen, zein, and wheat gluten), carbohydrates (e.g., alginate, chitosan, pullulan, guar-gum, tragacanth, inulin, cellulose, and dextrans), lipids (e.g., phospholipids), and animal/vegetal-origin synthetic polymers (e.g., polyvinyl alcohol and poly(ethylene) oxide). Bioactive compounds are then included *via* simple mixing into polymer spinning solution(s).^91^

However, at present, scale-up has a number of limitations. When spinnerets are arranged in arrays, the electrical field gets weaker from the edge to the center.^92^ The electrostatic force used to produce fiber from the spinneret reportedly then becomes a rate-limiting step.

An example scale-up however is the Nanospider electrospinning technology developed by Elmarco (www.elmarco.com). The process includes a rotating cylinder charged to 32 to 43 kV half-immersed in spinning dope.^93^ The liquid film is broken to form fibers collected above the tubular emitter.^72^ Nanolayr reported this scale-up of electrospinning.^94^

#### 4.1.2 Electrospraying

Electrospraying, or electro-hydrodynamic atomization, produces nanoparticles using high voltages, converting liquids to fine droplets that are stretched toward a ground electrode where the solvent is vaporized. The formation of nanocapsules is influenced by many parameters, including voltage, liquid velocity, collector distance, viscosity, density, concentration, pressure, and temperature.^95^

**Figure 10:**
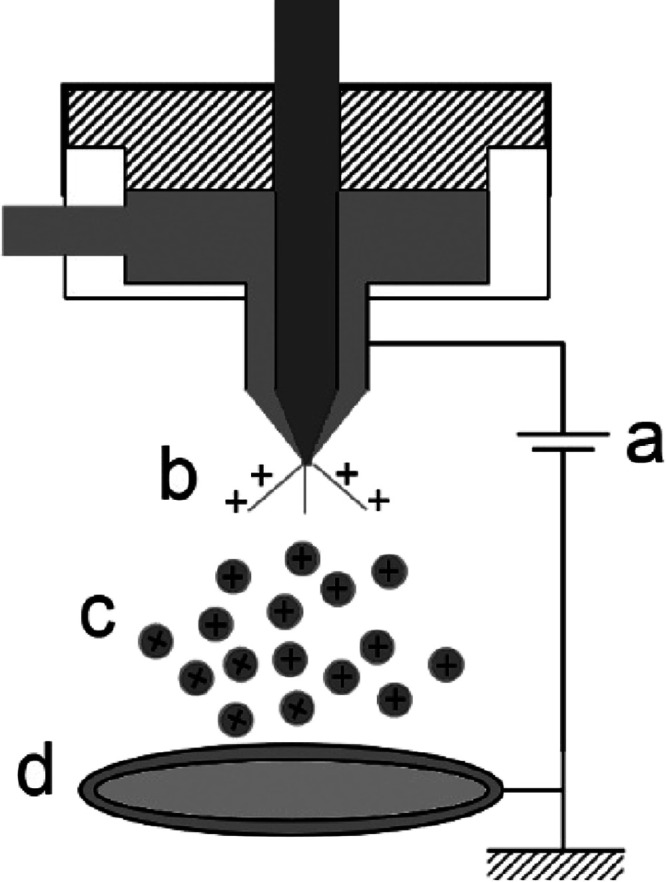
Conventional electrosprayer. (a) Power supply, (b) Taylor cone, (c) nanoparticle generation, and (d) nanoparticle collection.

Conventional electrospraying is shown schematically in Figure 10, where an electric field is used to split droplets into nanometer size by reducing surface tension *via* electric charge generation in a droplet.^96^ Particles are generated below the Taylor cone and collected on an Al plate. Process examples include production of wool keratin^97^ and zein films^98^ and encapsulation of curcumin in gelatin.^99^

A variation for this process involves electrospraying in solution, where charged droplets are collected in a cross-linking solution, typically calcium chloride. Two (2) polymer solutions are released concurrently using two (2) concentric needles of different diameters. This is known as electrospray electro-coextrusion.^100^ Active ingredients can be electrosprayed separately or together with a carrier polymer.^101^

Food examples include lycopene encapsulated in edible polymer matrices of dextran, chitosan, and whey protein concentrate.^102^

#### 4.1.3 Sustainability Lessons from Conventional Evaporation and Drying

Electrical process technologies involved in nanofood preparation, including electrospinning and electrospraying, comprise (1) ohmic heating, (2) electromagnetic activation, (3) evaporation or drying, and (4) high-pressure processing.

Lessons from conventional food technologies are available therefore from (3) evaporation or drying.

Drying, including evaporation, accounts for most thermal energy and electricity consumption within food processing. For milk powder production for example, this is *ca*. 44% of the total fuel consumption, equivalent to 616 MJ kg^–1^.^82^ Across the food industry, drying contributes *ca.* 25% of the total energy consumption.^103^ The environmental impact of drying is greater with longer operational times and/or higher temperatures.^103^ As is evident from Table 3, the impact depends on the type of drying and food ingredient(s), with an energy consumption similar to that for HBP.

A reported means to reduce energy consumption is to combine non-conventional drying methods, such as dehumidification, with conventional high-temperature drying. In this way, GWP can be reduced to *ca*. 48% and terrestrial acidification potential (TAP) can be reduced to *ca.* 59% for apricots by sequentially using osmotic dehydration and freeze-drying.^75^ Microwave drying of sardines reportedly reduces energy consumption by 55%^104^ and similarly for bananas.^105^ Combining infrared with hot air is demonstrated to accelerate drying.^106^ Combined microwave–vacuum drying has been reported for sea products,^107^ combined microwave–osmotic dehydration for pineapple,^108^ and combined microwave–freeze drying for jujube fruits.^109^ Other combinations have included combined infrared–freeze drying for mango^110^ and combined infrared–microwave drying for cake.^111^ Time-variant modulation has been applied to pulse-spouted microwave–vacuum drying,^112^ pulse-spouted bed microwave–freeze drying^113^ and microwave–freeze drying.^113^

To exemplify how the sustainability of conventional operations can be reduced, the energy consumption is taken. Two beneficial effects can be utilized. First, their industrial scale-up would optimize the heating per production unit, both per new design and by compactness (relatively less energy sources), the latter meaning less surface relative to the volume increase. Second, the energy source could be shifted toward renewables replacing the current gas or fossil fuel energy sources, either indirectly by using an electrical grid with high renewable share (could be an at-site microgrid) to change electricity to heat or by directly using renewably made electricity for heating. Both measures would allow a reduction in environmental impacts such as GWP, acidification, and depletion of abiotic resources.

#### 4.1.4 Conventional Drying Technologies

Conventional drying including air and sun is common because of low cost and simple operation.^9^ However, drawbacks include high energy demand, long drying time(s), and the potential for poor product quality from reduced sensory perception and nutritional value.^114,115^

The addition of an agent to lower water activity including humectants, e.g., sucrose, trehalose, or glycerol,^116^ results in osmotic dehydration by reducing the amount and mobility of water; however, this can induce negative effects on flavor, nutrition, and health.^117−119^ Taste alteration(s) and longer processing times have been reported.^120^

The interaction of electromagnetic waves with organic matter in electromagnetic drying can be selective to particular molecules rather than just heating the whole food.^9^ In this process, which is used to dry beef jerky^121^ and pork slices,^122^ energy is not always homogeneously distributed, causing “hot spots”. Other technologies include microwave and infrared^123^ and ultraviolet light, which is reported in some food applications,^124^ such as application in legume cakes.^111^ Rarely used are electron, X-ray, and gamma irradiation^125,126^ because these reportedly can ionize harmful microorganisms and damage valuable food components.^8^ Combined modes are reported including electroosmotic dewatering for tomato paste^127^ and electroosmotic-pressure dewatering for vegetable sludge.^128^ These combined conventional technologies can be useful for lessons in nanofood process development. For example, drying by infrared irradiation, combined with superheated steam, results in a lower GWP of 0.71 kg CO_2_-eq kg^–1^ for drying apricots^129^ as compared with drum-drying of apple pulp, which releases 2.67 kg CO_2_-eq kg^–1^.^74^

### 4.2 Thermally Driven Nanofood Technologies and Operation-Specific Sustainability

Lessons on operation-specific sustainability can be drawn from a comparison with conventional heating of thermally driven nanofood technologies based on nanospray drying for nanofood structure generation.

#### 4.2.1 Nanospray Drying

**Figure 11:**
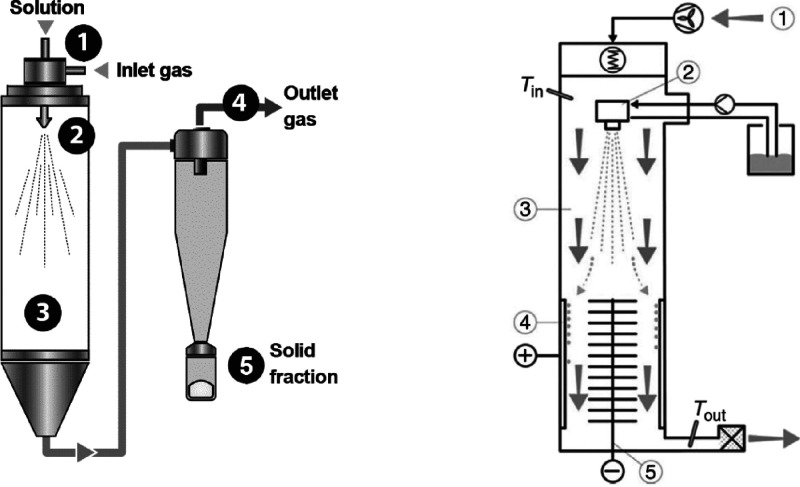
Conventional nanospray drying. Left: (1) atomization, (2) liquid nebulization in hot drying gas, (3) solvent evaporation, (4) product separation, and (5) solid product recovery. Right: nanospray drying adapted for nanoparticle production with same steps as for conventional drying (left) with added steps for (4) nanoparticle collection and (5) grounded electrodes.^133^ Reprinted with kind permission from Buchi Iberica S.L.U.

Nanospray drying uses a laminar drying gas that is projected toward a vibrating-mesh spray to form fine droplets, producing nanoparticles through an electrostatic precipitator. This process is adapted from conventional spray drying (Figure 11, left). Encapsulation is achieved by dissolving, emulsifying, or dispersing the core material in a carrier solution, including gums, carbohydrates, and/or proteins. Because solvent evaporation keeps droplet temperature low, the process can be used with heat-sensitive product(s).^130^ Nanoparticles can be made with relatively few process adaptions (Figure 11, right), with nanoparticle size and morphology targeted *via* process control. Nanospray drying is relatively “simple”, with short processing times. Costs are low compared with other drying technologies, such as freeze drying.^131^

Nanospray drying involves three (3) steps (Figure 11, left), namely, (1) atomization, (2) drying of nebulized droplets in a hot gas with solvent evaporation and corresponding particle formation, and (3) separation of dried product(s) using a cyclone in a conventional process (left) or particle collection in conventional processing (right).

Particle deposition on the chamber wall is reduced *via* the laminar flow of drying gas, vibrating mesh spraying, and electrostatic particle collectors (Figure 11, right).^132^

#### 4.2.2 Sustainability Lessons from Heating

Thermally driven process technologies involved in nanofood preparation and nanospraying involve (1) evaporation, (2) heating, and (3) electromagnetic activation.

Lessons from conventional food technologies are available therefore from (2) heating.

Thermal processing using steam and hot water is “standard” in the food industry. However, disadvantages include a necessary high investment in energy^134^ and a decrease in the sensory and nutritional value of the food. Environmental impact(s) from thermal processes depend significantly on treatment conditions. For example, the GWP for pasteurized milk ranges from *ca*. 0.114 to 0.427 kg CO_2_-eq L^–1^ and that for ultra-high temperature (UHT) processing ranges *ca*. 0.212 to 0.594 kg CO_2_-eq L^–1^, because UHT^77^ consumes greater energy. Thermal processing of milk and milk cream increases terrestrial acidification potential (TAP) because of energy consumption, together with subsequent wastewater generation.^135^ For orange juice processing, for example, pasteurization, blending, and cooling share *ca*. 20% of impacts,^136^ while for apple juice processing, the share is *ca*. 33%.^137^

For some foods, additional food operations add to the environmental impact, for example, pasteurization, molding, and salting or ripening used to produce cheese. Kim *et al.* quantified GWP and freshwater eutrophication potential (FEP) for cheddar cheese production to, respectively, 0.59 kg CO_2_-eq kg^–1^ and 0.43 × 10^–3^ kg P-eq kg^–1^.^138^ González-García *et al.* assessed Galician and mature cheese at, respectively, GWPs of 1.92 and 1.39 kg CO_2_-eq kg^–1^ and FEPs of 8 × 10^–3^ and 9.1 × 10^–3^ kg P-eq kg^–1^.^139^

Smoking is used as an additional operation to give food a particular flavor. This needs wood and fuel combustion. GWP accounts for 1.92 kg CO_2_-eq kg^–1^ for smoked cheese, together with an increase of 20% for GWP and 40% for TAP.^83^

#### 4.2.3 Conventional Drying Technology

Heat transfer occurs *via* convection, conduction, and/or radiation, with water (including steam), air, and oil as common transfer media. Heating inactivates pathogens, preserves foods,^140^ and improves digestibility and nutrient bioavailability.^141^ Thermal treatments can be classified as thermization or subpasteurization, with some pathogens developing resistance,^142^ pasteurization, e.g., for kiwi jam,^143^ fresh pasta,^144^ meat,^145^ and honey,^146^ ultra-high-temperature treatment, which is fast but can impart undesired flavor changes,^147^ sterilization, in which destroying all microorganisms, including most spores,^148^ can also result in unwanted sensory change(s),^148^ and ohmic heating^149^ for microorganism and enzyme inactivation,^150^ which is accompanied by a reduction in the size of protein aggregates and changes in protein morphology and physicochemistry^151^ and immunoreactivity.^151^ A combined mode of heating with refrigeration, followed by thermization and a second refrigeration, can potentially be used for cheese and/or protein whey.^152^

### 4.3 Cooling-Driven Nanofood Technologies and Operation-Specific Sustainability

#### 4.3.1 Desolvation of Protein-Based Nanoparticles

Lessons can be drawn for the desolvation of protein-based nanoparticles and nanocrystallization from the conventional processes of cooling, including cooling using mechanical and fluidic treatments. A desolvating agent can be used to precipitate biopolymers, e.g., proteins. Gelatin, albumin, collagen, milk proteins, silk protein, elastin, zein, gliadin, and soy protein have all been used.^153^ For example, Ipsen and Olsen described the conversion of α-lactalbumin to nanotubes.^154^ These gels are mechanically resistant, reversible, and pH-sensitive and are able to encapsulate vitamins and/or enzymes.^155^ A range of other morphologies are also possible.

#### 4.3.2 Nanocrystallization

**Figure 12:**
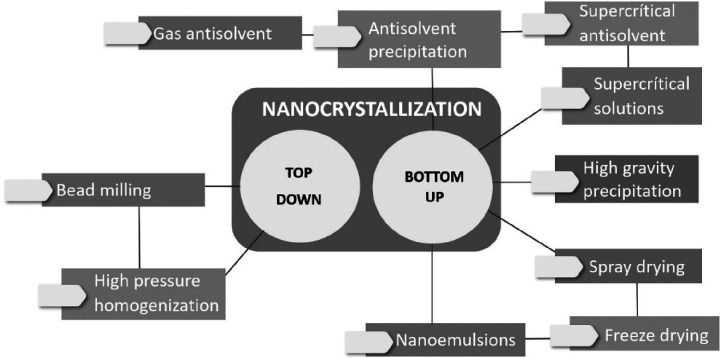
Top-down and bottom-up technology for nanocrystallization.

Nanocrystallization refers to precipitation (bottom-up) or size reduction of larger crystals (top-down) as a means to define nanocrystal size^156−158^ and can involve a range of techniques, some of which involve cooling (Figure 12).

**Figure 13:**
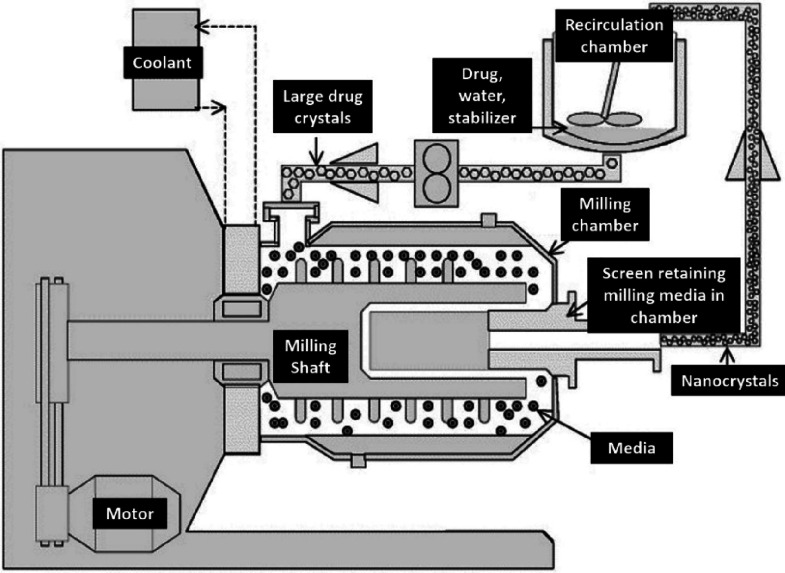
Medium milling. Adapted from Merisko-Liversidge *et al*.^160^ with kind permission from Elsevier Ltd. Copyright 2003.

Commonly, top-down processing involves wet milling, including (1) bead milling (Nanosystems-Alkermes^159^), combined with (2) high-pressure homogenization and smartCrystal technology (fast and smaller size production of nanocrystals than first-generation top-down or bottom-up methods). In bead milling, a macro-suspension is produced when a powder is dispersed in a stabilizer solution that is recirculated several times through the bead-mill chamber (Figure 13). A coolant also reduces the temperature during milling. Particle size reduction is a result of shear forces produced by moving beads and the collision of particles. Disadvantages include the production of low nanometer particles and the need to remove all solvent traces.

High-pressure homogenization reduces particle size by shear force(s), particle collisions, and cavitation. Two (2) variants are reported.^161^ Microfluidization collides two (2) crystal suspensions under a pressure of 1700 bar. Piston-gap homogenization forces through a small nozzle, creating a sudden pressure gradient. Disadvantages are the need for micronized starting material and long runtimes.^157^

Combined technologies reportedly have practical promise to overcome these limitations. NANOEDGE technology,^162^ H42 technology,^163^ and H96 technology^164^ integrate microprecipitation, spray drying, and pearl milling. H69 methodology leverages smartCrystal technology with cavitation taking place concurrently with particle formation. This is called cavi-precipitation. Collision and shear forces stop nucleation and prevent further crystal growth. Examples include the microfluidizer and EmulsiFlex C5 from Avestin.^165^

#### 4.3.3 Sustainability Learning from Cooling

Cooling nanofood process technologies involve (1) heating, (2) cooling, (3) mechanical treatment, (4) electromagnetic activation, and (5) evaporation.

Lessons from conventional food technologies are available from (2) cooling and (4) electromagnetic activation.

Food process freezing at temperatures of <−18 °C is used to secure high-quality frozen goods that maintain original flavor, color, and nutrition. These are commonly preceded by pre-freezing. Depending on the nature of raw materials, pre-freezing is accompanied by other secondary operations, including washing, peeling and cutting, sorting, or blanching.^166^ Freezing exhibits lower LCA as compared with raw food production.^166^ Freezing has an environmental impact of similar GWP to drying and heating. Ilari *et al.* reported for freezing green beans a GWP of 0.7 kg of CO_2_-eq kg^–1^, abiotic depletion potential of 9.5 MJ-eq kg^–1^, fresh water ecotoxicity of 0.2 kg 1,4-DB-eq kg^–1^, and marine aquatic ecotoxicity of 312 kg 1,4-DB-eq kg^–1^.^166^ For broccoli, the overall environmental impact increased by 25 to 50% when compared with fresh food, with the exception of the eutrophication potential, which decreased.^84^ Abiotic depletion potential for frozen broccoli was 1.71 × 10^–2^ kg Sb-eq kg^–1^, acidification potential was 9.32 × 10^–3^ kg SO_2_-eq kg^–1^, eutrophication potential was 4.49 × 10^–3^ kg PO_4_-eq kg^–1^, GWP was 2.64 kg CO_2_-eq kg^–1^, and photochemical oxidation potential was 7.71 × 10^–4^ kg C_2_H_4_-eq kg^–1^.^84^

#### 4.3.4 Sustainability Lessons from Electromagnetic Activation

Non-thermal processing, including high pressure (HP), ionizing irradiation, and pulsed electric field (PEF), provides alternatives to thermal treatment, together with a claimed reduction in the negative impact on food quality with increased energy efficiency.^167^

However, there is inconsistent reporting of efficiency as exemplified by Davis *et al.*(168) who report an HP of 0.23 MJ L^–1^ and PEF of 0.17 MJ L^–1^ to be more energy-efficient than thermal treatment of 0.26 MJ L^–1^ for carrot juice, whereas Aganovic *et al.* report 3 to 5× greater energy demand for nonthermal processing with 0.72 MJ L^–1^ HP and 0.43 MJ L^–1^ PEF compared with thermal treatment of 0.14 MJ L^–1^for tomato and watermelon juice.^134^

It is concluded therefore that caution must be exercised with generalized statements for classes of food technology; rather, performance will necessarily be a function of the food and technology used within a given class.

For GWP, however, the trend is clearer. The GWP (CO_2_-eq kg^–1^ kg) ratios for thermal treatment to HP for tomato salsa,^167^ ready-to-eat meal,^169^ and milk^170^ are reportedly, respectively, infinite, 2.7, and 8.7. Valsasina *et al.* confirmed that HP has 83% lower carbon footprint for milk homogenization, as compared with thermal treatment.^170^

Mikhaylin *et al.* report applying bipolar membrane electrodialysis with ultrafiltration with 10% lower environmental impact, as compared with conventional acid–base processing that uses hazardous chemicals.^171^ Vauchel *et al.* used ultrasound for polyphenol extraction from chicory grounds, reducing the environmental impact by up to 25% in 16 categories.^73^

#### 4.3.5 Cooling Including Mechanical and Fluidic Treatments

A further variation on cooling is those processes involving mechanical and fluidic treatments. Cooling inhibits undesired chemical reactions including fat oxidations or polymeric hydrolysis by reducing molecular mobility and reaction rate(s).^172^ However, cooling can cause undesired structural damage(s), including crystallization and cell destruction, with the release of intracellular contents.^173^ Freezing can be combined with osmotic drying,^174^ as reported for mango^175^ and tomatoes.^176^

Mechanical treatment uses applied force for reversible, e.g., sorting, or irreversible, e.g., milling, material deformation.^177^ High static or dynamic pressure(s) changes the distribution of compounds in foods and renders microbes non-viable^178,179^ and lowers the energy demand for added value, e.g., in artificial meat production.^180^

Osmotic pressure allows removal of water from food, together with introduction of solutes,^181^ e.g., kiwi slices.^182^ Vacuum impregnation brings fluids into food and removes deleterious reactive gases, including oxygen.^183^ Drying rates are faster than by drying alone, canning, freezing, or frying pre-treatment. Combined with osmotic dehydration,^183^ gases are removed from capillary pores,^184^ and the osmotic solution flows into the pores.^185−187^ For Spanish cured ham, process times^188^ were significantly reduced, and calcium-fortified pineapple snacks resulted in an improved sensory profile with long storage.^189^ Drawbacks however included poor mass transfer control and practical obstacles to the reuse of hypertonic solution(s).^190^

Ultrasound technology is reportedly used for fluid transport in meat tenderization, either continuously or pulsed-applied^191^*via* pressure, temperature, or volumetric compression/expansion changes(s).^191^ Combined with osmotic dehydration, ultrasound creates microscopic channels in tissues to ease gas–osmotic solution exchange,^192^ e.g., in potato^193^ and Malay apple.^194^

### 4.4 Dispersion-Driven Nanofood Technologies and Operation-Specific Sustainability

Lessons can be drawn for nanoemulsions and nanoliposome formation from the conventional processes of extraction and postprocessing operations.

#### 4.4.1 Nanoemulsions

Tadros *et al.* classified nanoemulsion techniques according to energy demand into high-energy and low-energy ones.^195^ Nanoemulsions need to be made with control over droplet size and size distribution.^196^ The choice of technique depends on ingredients and properties such as stability. Nanoemulsification uses high-pressure homogenizers with 99.8% of energy spent to produce heat. Therefore, chemical degradation of food materials can occur.^197^ Additionally, entrapment of air needs to be avoided to prevent unwanted foam formation.^198^

Operational integration is as important as for other food process technologies. Nanoemulsification is usually carried out in multiple steps, starting from separate oil and water solutions that are transferred in a high-speed colloid mill at revolutions of up to 30,000 rpm with controlled accelerations and decelerations to produce high tangential forces. Different internal shear regions are utilized. This avoids coalescence.^199^ Microfluidizers and ultrasonic homogenizers are used for pre-homogenization, while high-pressure homogenizers deliver pre-emulsions.^200^

**Figure 14:**
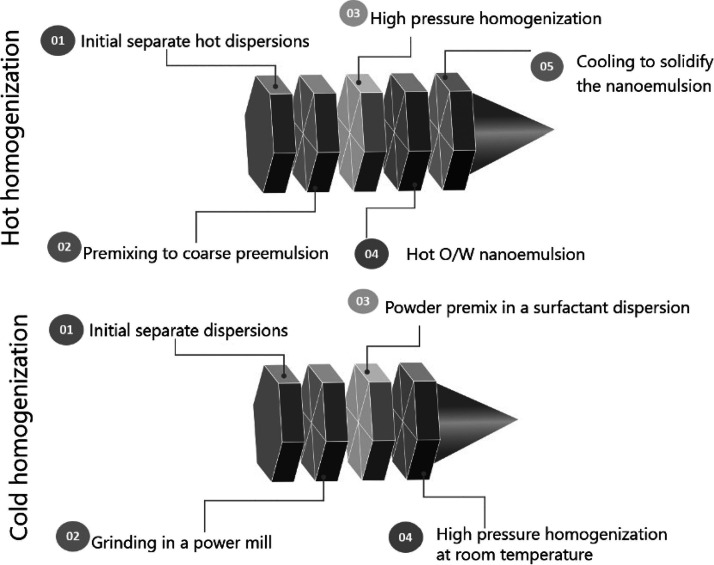
Flow for hot^201^ and cold^32a^ pre-homogenization in the production of nanoemulsions.

In addition to energy demand(s), homogenization is carried out in either a hot or cold environment (Figure 14). This is governed by the melting point of the hydrophobic phase, viscosity to prevent degradation of functional materials and heterogeneous distribution of the phases, and complex crystallization during cooling.^32a^

**Figure 15:**
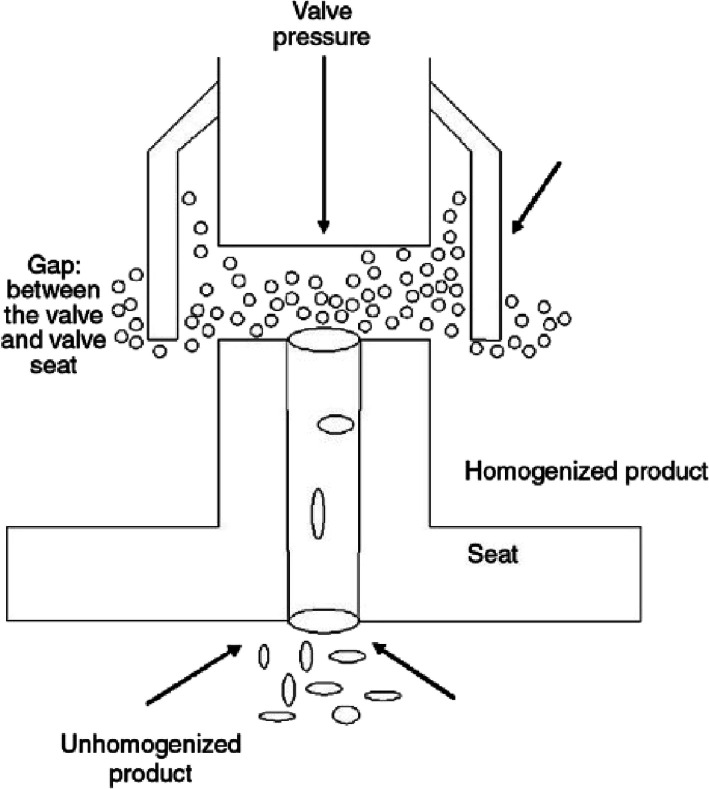
High-pressure valve homogenizer. Reprinted with kind permission of Elsevier Ltd.^205^ Copyright 2015.

Following pre-homogenization, a second homogenization step is used to reduce droplet diameter to a nano-size range. High-pressure valve homogenizers with a gap of 10 to 100 μm are used at pressures in the range of 100 to 500 MPa together with refrigeration for cooling (Figure 15).^202^ An example is that microfluidizers pre-homogenize emulsions under pressures of 35 to 140 MPa and then pump the fluid to a microchannel section, generating a flow of high turbulence.^130^ At the outlet, the fluid is ejected as elongational flow for adsorption of the emulsifier to form interfaces.^203^ High-pressure valve homogenization is used to generate essential oils and to encapsulate bioactive ingredients.^204^

Ultrasound is also reportedly used to reduce droplet diameter. High-intensity ultrasonication produces up to 100 nm nanodroplets operating at a frequency of 16 to 100 kHz and a power density of 10 to 1000 W cm^–2^. As stability is improved, less emulsifying agent is needed compared to preparations made with high-pressure homogenizers.^206^ Sizing sonochemical reactors to the industrial scale is a practical challenge because of physical limitations of the technology. This technology has the potential, however, for reduced production costs and equipment contamination compared with microfluidizers.^207^ Pascual-Pineda *et al.* reportedly prepared a paprika oleoresin nanoemulsion^208^ using these techniques.

In opposition to high-energy methods, low-energy methodologies are based on phase inversion by changing the hydrophilic–lipophilic balance *via* either temperature or composition.^200^ However, this technology requires a greater concentration of surfactants and greater dispersity of droplets. Phase inversion is achieved by adding water to a stirred oil–surfactant mixture to change the O/W ratio toward an excess of water, reversing dispersity to give an oil-in-water suspension.^197^ Alternatively, heating to above the temperature for phase inversion changes the solubility of the surfactant, inducing a phase transition that changes the emulsion type.^209^ Rapid cooling then freezes this phase change.^210^

In membrane emulsification, the dispersed phase is pressed through a membrane that forms emulsions directly, or this technique can be used to premix.^211^ Major process parameters include membrane type, pore size, cross-flow speed, pressure, and type of emulsifier.

#### 4.4.2 Nanoliposomes

Conventional methodologies for producing nanoliposomes include (1) thin-film hydration, (2) solvent injection, (3) detergent removal, and (4) reverse phase. Methodology (1) is based on aqueous rehydration of thin phospholipid films initially dissolved in an organic solvent. Once the solvent is evaporated, the film is composed of the encapsulates. The concept for methodology (2) involves the solubilization of the hydrophobic fraction in an aqueous system using a solvent, e.g., ethanol. In methodology (3), an excess of surfactants is eliminated once micelles are produced using dialysis. In methodology (4), the dissolution of lipids in a solvent to form a water-in-oil emulsion is utilized. Liposome formation is preceded by solvent removal in the aqueous dispersion.

These four (4) methodologies have drawbacks including (1) the large size of liposomes, (2) remainder of solvent traces, (3) destabilization induced by temperature changes, and (4) low reproducibility.^212^

Supercritical fluid technology, dual asymmetric centrifugation, membrane contactor technology, cross-flow filtration technology, and freeze-drying technology have practical promise to obviate these drawbacks. The goal is to minimize destabilization, as well as degradation. This can be achieved by controlling the composition, temperature and pH, nature of the materials, ionic strength, and sensitivity to light or oxygen.^213^

**Figure 16:**
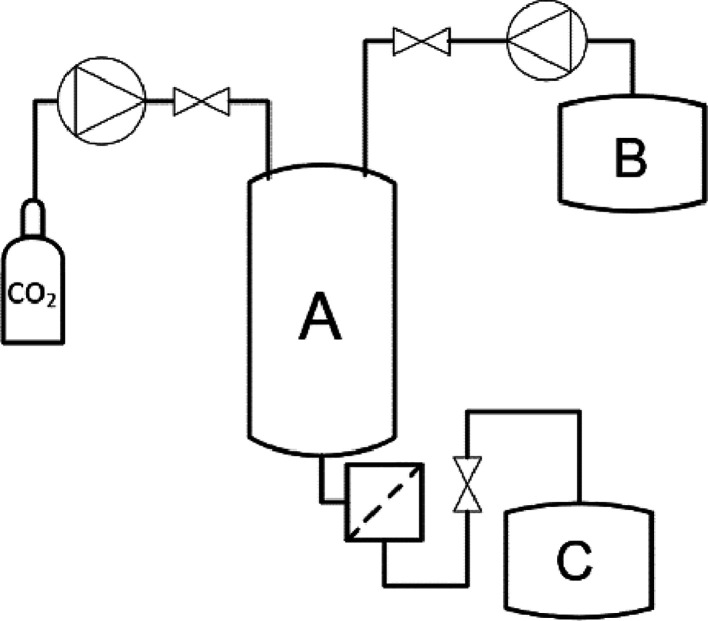
Scheme for supercritical antisolvent technology for liposome preparation. (A) Supercritical reactor (temperature/pressure), (B) starting suspension, e.g., lecithin and cholesterol in ethanol, and (C) organic solvent trap.

In an alternative method, the dissolution of lipids, e.g., phospholipids and cholesterol, in supercritical CO_2_ (scCO_2_) allows for a supersaturation state that facilitates precipitation to ultrafine particles following the release of CO_2_. Liposomes are obtained by concurrently adding an aqueous phase.^214^ A variant is supercritical reverse phase evaporation for liposome production using scCO_2_ as a solvent for lipids (Figure 16).^215^

Dual asymmetric centrifugation has double-axis rotation, one centering vials and the other a centrifuge. Material is pushed in an outward direction, while concurrently, material is pushed in the opposite direction because of adhesion, which reportedly works well with viscous material(s).^216^ This methodology allows the compactness of equipment, good reproducibility, the formation of small liposomes without the need of additional operations, and high entrapment efficiency. A minimum phospholipid concentration is required for sufficient viscosity.^212^

Membrane contactors disperse lipid and water phases to form liposomes. While the aqueous phase is pumped through the membrane contactor, the lipid phase is pumped perpendicularly, permeating the internal pores of membrane fibers of the contactor. Liposomes are formed “spontaneously” once both phases meet. Advantages include ready scalability, reproducibility, small size dispersion, and high encapsulation efficiency.^217^

Cross-flow filtration technology pumps the mixed micelle solution through a pressurized membrane. The pressure acts to remove the surfactant. Advantages are fast production and filtrate recycling to minimize waste.^218^

Freeze drying uses cryoprotectants, such as carbohydrates, to facilitate transition of liposome suspension to liposomes, with sizes <200 nm.^219,220^

#### 4.4.3 Sustainability Lessons from Post-Processing (Including Extraction)

Dispersion-driven nanofood process technologies involve (1) heating, (2) cooling, (3) mechanical treatment, (4) ultrasonication, and (5) evaporation. Lessons that can be learned from conventional food technologies are available from each of (1) through (4). Lesson(s) and understanding are additionally available from post-processing, i.e., extraction, as this is a further dispersive process. Cleaning commonly requires hot water, which increases energy, costs, and further environmental impact.

Extraction of food waste products can reduce and recover resources. Reported extraction technologies include ultrasound-assisted, microwave-assisted, enzyme-assisted, bioreactor-assisted, and/or high-pressure homogenizer technologies. Food waste generates some 113 Mt of CO_2_ per annum^221^ from resources used for biomass production, including water, fertilizer, pesticides, feed or seed, and energy.^222^ An outcome from this recovery is bioactive compounds,^223^ including health-promoting additives and nutritional supplements,^224^ or biofuels. Al-Dhabi *et al.* reported an ultrasound process that was claimed to be economically feasible and have a low energy demand for extraction of phytochemicals from exhausted coffee grounds.^225^ Using ultrasound technology, Guandalini *et al.* demonstrated an improved pectin extraction of mango peel of 50% and, importantly, claimed that the pectin quality was maintained.^226^ With microwave processing combined with deep eutectic solvents,^227^ Pal and Jadeja conducted extraction of phenolic compounds from ripe mango.^228^ Plazzotta *et al.* extracted valuable flavonoids and anthocyanins from frozen peach waste in <1 min *via* microwave technology.^229^ Operating this technology with recycling, Escribà *et al.*(230) transformed vegetable oil and food fats into allyl esters^231^ for use as ovicides against *Cydia pononella* L.^232^ When strong oxidants, e.g., potassium dichromate, are used, cleaning effluent that forms a wastewater output impacts freshwater eutrophication potential (FEP).

#### 4.4.4 Post-Processing Operation

Cleaning, extraction, and decontamination remove deposits in heat exchangers, evaporators, and pipelines and increase water and chemical consumption.^233^ Plasma, acknowledged as the fourth state of matter, is reportedly being used to decontaminate foods^9^*via* inactivation of microorganisms and can be used to remove biofilms, bacterial spores, fungi, and bacteria^234,235^ with bacon,^236^ cheese,^237^ jam,^238^ and squid^239^ being reported applications. Plasma can also boost the flavor and aroma of foods.^240^

## 5 Conclusions

Nanostructures provide a new and practical opportunity for food products *via* supramolecular architecture(s) to encapsulate bioactive molecules and to control targeted release.

Acceptance of these products in food processing is dependent on compliance with legislation and technological and economic sustainability. The economy of scale will also be important to ensure sustainability.

Only a limited number of nanofood processes have matured to industrial, or large pilot-scale, production. A sustainable methodology is therefore necessary to underpin developments in nanofood technology. It was found that this sustainable methodology must begin at the initial or laboratory scale.

Predictive sustainability assessment(s), including exploratory *ex ante* and anticipatory life cycle assessment(s), have been widely used to develop emerging technologies in chemistry and mining. To date, these have surprisingly not been used in food processing. It was hypothesized therefore that a methodology could be developed from sustainability assessments for conventional food technologies and applied practically to design for nanofood technology.

It was found that unit operations shared between conventional and emerging processes were a sufficiently pragmatic means for the development of sustainability. This is because there is a limited number of essential engineering operations including heating, drying, and post-processing, which determine the majority of conventional and nanofood technologies. Sustainability for these conventional processes is also well documented.

We conclude that this provides a quantitative means to incorporate sustainability during early process design for nanostructured foods. A major difficulty, however, to developing a generalized and quantitative methodology is that sustainability is unit operation-specific and food product-specific. Further development is therefore needed to create a transferable sustainability methodology.

However, the present findings will be of interest to food researchers and engineers, as well as manufacturers of process equipment.

## References

[ref1] Monteiro C. A.; Moubarac J. C.; Levy R. B.; Canella D. S.; da Costa Louzada M. L.; Cannon G. Household availability of ultra-processed foods and obesity in nineteen European countries. Public Health Nutr. 2018, 21, 18–26. 10.1017/S1368980017001379. 28714422 PMC10260838

[ref2] Araújo R. G.; González-González R. B.; Martinez-Ruiz M.; Coronado-Apodaca K. G.; Reyes-Pardo H.; Morreeuw Z. P.; Oyervides-Muñoz M. A.; Sosa-Hernández J. E.; Barceló D.; Parra-Saldívar R.; Iqbal H. M. N. Expanding the Scope of Nanobiocatalysis and Nanosensing: Applications of Nanomaterial Constructs. ACS Omega 2022, 7, 32863–32876. 10.1021/acsomega.2c03155. 36157779 PMC9494649

[ref3] Biswas R.; Alam M.; Sarkar A.; Haque M. I.; Hasan M. M.; Hoque M. Application of nanotechnology in food: Processing, preservation, packaging and safety assessment. Heliyon 2022, 8, e11795 10.1016/j.heliyon.2022.e11795. 36444247 PMC9699984

[ref4] Gupta I.; Cherwoo L.; Bhatia R.; Setia H. Biopolymers: Implications and application in the food industry. Biocatal. Agric. Biotechnol. 2022, 46, 102534 10.1016/j.bcab.2022.102534.

[ref5] Ozogul Y.; Karsli G. T.; Durmuş M.; Yazgan H.; Oztop H. M.; McClements D. J.; Ozogul F. Recent developments in industrial applications of nanoemulsions. Adv. Colloid Interface Sci. 2022, 304, 102685 10.1016/j.cis.2022.102685. 35504214

[ref6] A.S., Foust; Wenzel L.A.; Clump C.W.; Maus L.; Andersen L.B.; Bryce L. in Principles of unit operations; 2nd edition, John Wiley & Sons, New York, 1980, pp. 768.

[ref7] Hessel V.; Escribà-Gelonch M.; Bricout J.; Tran N. N.; Anastasopoulou A.; Ferlin F.; Valentini F.; Lanari D.; Vaccaro L. Quantitative sustainability assessment of flow chemistry–From simple metrics to holistic assessment. ACS Sustainable Chem. Eng. 2021, 9, 9508–9540. 10.1021/acssuschemeng.1c02501.

[ref8] Meijer G. W.; Lähteenmäki L.; Stadler R. H.; Weiss J. Issues surrounding consumer trust and acceptance of existing and emerging food processing technologies. Crit. Rev. Food Sci. Nutr. 2021, 61, 97–115. 10.1080/10408398.2020.1718597. 32003225

[ref9] Qiu L.; Zhang M.; Tang J.; Adhikari B.; Cao P. Innovative technologies for producing and preserving intermediate moisture foods: A review. Food Res. Int. 2019, 116, 90–102. 10.1016/j.foodres.2018.12.055. 30717022

[ref10] McClements D. J. Nanoscale nutrient delivery systems for food applications: Improving bioactive dispersibility, stability, and bioavailability. J. Food Sci. 2015, 80, N1602–N1611. 10.1111/1750-3841.12919. 26073042

[ref11] aAlAli M.; Alqubaisy M.; Aljaafari M. N.; AlAli A. O.; Baqais L.; Molouki A.; Abushelaibi A.; Lai K.-S.; Lim S.-H. E. Nutraceuticals: Transformation of conventional foods into health promoters/disease preventers and safety considerations. Molecules 2021, 26, 2540 10.3390/molecules26092540. 33925346 PMC8123587

[ref12] Aditya N. P.; Ko S. Solid lipid nanoparticles (SLNs): Delivery vehicles for food bioactives. RSC Adv. 2015, 5, 30902–30911. 10.1039/C4RA17127F.

[ref13] Plucinski A.; Lyu Z.; Schmidt B. V. K. J. Polysaccharide nanoparticles: from fabrication to applications. J. Mater. Chem. B 2021, 9, 7030–7062. 10.1039/D1TB00628B. 33928990

[ref14] aMartins J. T.; Bourbon A. I.; Pinheiro A. C.; Fasolin L. H.; Vicente A. A. Protein-based structures for food applications: from macro to nanoscale. Front. Sustain. Food Syst. 2018, 2, 77 10.3389/fsufs.2018.00077.

[ref15] A., Das; Sen C. K. in Nutraceutical and Functional Food Regulations in the United States and Around the World (2nd Edition) Bagchi D. Ed.; Academic Press: San Diego, 2014, pp. 13–39.

[ref16] Patidar A.; Thakur D. S.; Kumar P.; Verma J. A review on novel lipid based nanocarriers. Int. J. Pharm. Pharm. Sci. 2010, 2, 30–35. 10.1016/B978-0-12-405870-5.00002-5.

[ref17] National Research Council (US) Committee on Diet and Health. Diet and Health: Implications for Reducing Chronic Disease Risk; National Academies Press (US) 1989, 10, 17226/1222. 25032333

[ref18] da Silva Santos V.; Badan Ribeiro A. P.; Santana M. H. A. Solid lipid nanoparticles as carriers for lipophilic compounds for applications in foods. Food Res. Int. 2019, 122, 610–626. 10.1016/j.foodres.2019.01.032. 31229120

[ref19] aTamjidi F.; Shahedi M.; Varshosaz J.; Nasirpour A. Nanostructured lipid carriers (NLC): A potential delivery system for bioactive food molecules. Innovative Food Sci. Emerging Technol. 2013, 19, 29–43. 10.1016/j.ifset.2013.03.002.

[ref20] Espitia P. J. P.; Fuenmayor C. A.; Otoni C. G. Nanoemulsions: Synthesis, characterization, and application in bio-based active food packaging. Compr. Rev. Food Sci. Food Saf. 2019, 18, 264–285. 10.1111/1541-4337.12405. 33337016

[ref21] McClements D. J.; Rao J. Food-grade nanoemulsions: Formulation, fabrication, properties, performance, biological fate, and potential toxicity. Crit. Rev. Food Sci. Nutr. 2011, 51, 285–330. 10.1080/10408398.2011.559558. 21432697

[ref22] Reyes Y.; Hamzehlou S.; Leiza J. R. Ostwald ripening in nano/miniemulsions in the presence of two costabilizers as revealed by molecular dynamics simulations. J. Mol. Liq. 2021, 335, 116152 10.1016/j.molliq.2021.116152.

[ref23] Acosta E. Bioavailability of nanoparticles in nutrient and nutraceutical delivery. Curr. Opin. Colloid Interface Sci. 2009, 14, 3–15. 10.1016/j.cocis.2008.01.002.

[ref24] Aswathanarayan J. B.; Vittal R. R. Nanoemulsions and their potential applications in food industry. Front. Sustain. Food Syst. 2019, 3, 95 10.3389/fsufs.2019.00095.

[ref25] McClements D. J. Nanoemulsions *versus* microemulsions: Terminology, differences, and similarities. Soft Matter 2012, 8, 1719–1729. 10.1039/C2SM06903B.

[ref26] aLivney D. Y. Nanostructured delivery systems in food: latest developments and potential future directions. Curr. Opin. Food Sci. 2015, 3, 125–135. 10.1016/j.cofs.2015.06.010.

[ref27] aReza Mozafari M.; Khosravi-Darani K.; Gokce Borazan G.; Cui J.; Pardakhty A.; Yurdugul S. Encapsulation of food ingredients using nanoliposome technology. Int. J. Food Prop. 2008, 11, 833–844. 10.1080/10942910701648115.

[ref28] Patel A. R.; Velikov K. P. Colloidal delivery systems in foods: A general comparison with oral drug delivery. LWT--Food Sci. Technol. 2011, 44, 1958–1964. 10.1016/j.lwt.2011.04.005.

[ref29] M., Gupta; Sharma V.; Chauhan N. S. in Nano- and Microscale Drug Delivery Systems (Ed.: A. M., Grumezescu), Elsevier, 2017, pp. 197–228, 10.1016/B978-0-323-52727-9.00011-X.

[ref30] N., Singh; Joshi A.; Toor A. P.; Verma G. in Nanostructures for Drug Delivery (Eds.: E., Andronescu; Grumezescu A. M.), Elsevier, 2017, pp. 865–886, 10.1016/B978-0-323-46143-6.00027-0.

[ref31] Müller R. H.; Radtke M.; Wissing S. A. Solid lipid nanoparticles (SLN) and nanostructured lipid carriers (NLC) in cosmetic and dermatological preparations. Adv. Drug Delivery Rev. 2002, 54, S131–S155. 10.1016/S0169-409X(02)00118-7. 12460720

[ref32] aMehnert W.; Mäder K. Solid lipid nanoparticles: Production, characterization and applications. Adv. Drug Delivery Rev. 2001, 47, 165–196. 10.1016/s0169-409x(01)00105-3. 11311991

[ref33] Das S.; Chaudhury A. Recent advances in lipid nanoparticle formulations with solid matrix for oral drug delivery. AAPS PharmSciTech. 2011, 12, 62–76. 10.1208/s12249-010-9563-0. 21174180 PMC3066374

[ref34] Müller R. H.; Mäder K.; Gohla S. Solid lipid nanoparticles (SLN) for controlled drug delivery - A review of the state of the art. Eur. J. Pharm. Biopharm. 2000, 50, 161–177. 10.1016/s0939-6411(00)00087-4. 10840199

[ref35] Bourbon A. I.; Pereira R. N.; Pastrana L. M.; Vicente A. A.; Cerqueira M. A. Protein-based nanostructures for food applications. Gels 2019, 5, 9 10.3390/gels5010009. 30813359 PMC6473444

[ref36] aMohammad A. W.; Suhimi N. M.; Aziz A. G. K. A.; Jahim J. M. Process for production of hydrolysed collagen from agriculture resources: Potential for further development. J. Appl. Sci. 2014, 14, 1319–1323. 10.3923/jas.2014.1319.1323.

[ref37] aMcClements D. J.; Decker E. A.; Park Y.; Weiss J. Structural design principles for delivery of bioactive components in nutraceuticals and functional foods. Crit. Rev. Food Sci. Nutr. 2009, 49, 577–606. 10.1080/10408390902841529. 19484636

[ref38] Sağlam D.; Venema P.; van der Linden E.; Vries R. Design, properties, and applications of protein micro- and nanoparticles. Curr. Opin. Colloid Interface Sci. 2014, 19, 428–437. 10.1016/j.cocis.2014.09.004.

[ref39] Martins J. T.; Ramos Ó. L.; Pinheiro A. C.; Bourbon A. I.; Silva H. D.; Rivera M. C.; Cerqueira M. A.; Pastrana L.; Malcata F. X.; González-Fernández A.; Vicente A. A. Edible bio-based nanostructures: Delivery, absorption and potential toxicity. Food Eng. Rev. 2015, 7, 491–513. 10.1007/s12393-015-9116-0.

[ref40] E.H., Song; Shang J.; Ratner D.M. in Polymer Science: A Comprehensive Reference; (Eds.: K., Matyjaszewski; Möller M.), Elsevier: Amsterdam, 2012, pp. 137–155, 10.1016/B978-0-444-53349-4.00246-6.

[ref41] aZong A.; Cao H.; Wang F. Anticancer polysaccharides from natural resources: A review of recent research. Carbohydr. Polym. 2012, 90, 1395–1410. 10.1016/j.carbpol.2012.07.026. 22944395

[ref42] Yang J.; Han S.; Zheng H.; Dong H.; Liu J. Preparation and application of micro/nanoparticles based on natural polysaccharides. Carbohydr. Polym. 2015, 123, 53–66. 10.1016/j.carbpol.2015.01.029. 25843834

[ref43] Cui R.; Zhu F. Ultrasound modified polysaccharides: A review of structure, physicochemical properties, biological activities and food applications. Trends Food Sci. Technol. 2021, 107, 491–508. 10.1016/j.tifs.2020.11.018.

[ref44] Chaves M. A.; Baldino L.; Pinho S. C.; Reverchon E. Supercritical CO2 assisted process for the production of mixed phospholipid nanoliposomes: Unloaded and vitamin D3-loaded vesicles. J. Food Eng. 2022, 316, 110851 10.1016/j.jfoodeng.2021.110851.

[ref45] Chaves M. A.; Franckin V.; Sinigaglia-Coimbra R.; Pinho S. C. Nanoliposomes coencapsulating curcumin and vitamin D3 produced by hydration of proliposomes: Effects of the phospholipid composition in the physicochemical characteristics of vesicles and after incorporation in yoghurts. Int. J. Dairy Technol. 2021, 74, 107–117. 10.1111/1471-0307.12729.

[ref46] Gülseren İ.; Guri A.; Corredig M. Encapsulation of tea polyphenols in nanoliposomes prepared with milk phospholipids and their effect on the viability of HT-29 human carcinoma cells. Food Dig. 2012, 3, 36–45. 10.1007/s13228-012-0019-8.

[ref47] Sebaaly C.; Jraij A.; Fessi H.; Charcosset C.; Greige-Gerges H. Preparation and characterization of clove essential oil-loaded liposomes. Food Chem. 2015, 178, 52–62. 10.1016/j.foodchem.2015.01.067. 25704683

[ref48] Mohammadi M.; Ghanbarzadeh B.; Hamishehkar H. Formulation of nanoliposomal vitamin D3 for potential application in beverage fortification. Adv. Pharm. Bull. 2014, 4, 569–575. 10.5681/apb.2014.084. 25671191 PMC4312407

[ref49] Xia S.; Tan C.; Zhang Y.; Abbas S.; Feng B.; Zhang X.; Qin F. Modulating effect of lipid bilayer–carotenoid interactions on the property of liposome encapsulation. Colloids Surf., B 2015, 128, 172–180. 10.1016/j.colsurfb.2015.02.004. 25747311

[ref50] Abdollahzadeh M.; Elhamirad A. H.; Shariatifar N.; Saeidiasl M.; Armin M. Effects of nano-chitosan coatings incorporating with free/nano-encapsulated essential oil of Golpar (Heracleum persicum L.) on quality characteristics and safety of rainbow trout (Oncorhynchus mykiss). Int. J, Food Microbiol. 2023, 385, 109996 10.1016/j.ijfoodmicro.2022.109996. 36403331

[ref51] Liu W.; Tian M.; Kong Y.; Lu J.; Li N.; Han J. Multilayered vitamin C nanoliposomes by self-assembly of alginate and chitosan: Long-term stability and feasibility application in mandarin juice. LWT--Food Sci. Technol. 2017, 75, 608–615. 10.1016/j.lwt.2016.10.010.

[ref52] Hosseini S. F.; Soofi M.; Rezaei M. Enhanced physicochemical stability of ω-3 PUFAs concentrates-loaded nanoliposomes decorated by chitosan/gelatin blend coatings. Food Chem. 2021, 345, 128865 10.1016/j.foodchem.2020.128865. 33601664

[ref53] Gonçalves R. F. S.; Martins J. T.; Abrunhosa L.; Vicente A. A.; Pinheiro A. C. Nanoemulsions for Enhancement of Curcumin Bioavailability and Their Safety Evaluation: Effect of Emulsifier Type. Nanomaterials (Basel) 2021, 11, 815 10.3390/nano11030815. 33806777 PMC8004751

[ref54] Han W.; Liu T.-X.; Tang C.-H. Use of oligomeric globulins to efficiently fabricate nanoemulsions: Importance of enhanced structural stability by introducing trehalose. Food Hydrocolloids 2023, 141, 108695 10.1016/j.foodhyd.2023.108695.

[ref55] Medeiros A. K. D. O. C.; de Carvalho Gomes C.; de Araújo Amaral M. L. Q.; de Medeiros L. D. G.; Medeiros I.; Porto D. L.; Aragão C. F. S.; Maciel B. L. L.; de Araújo Morais A. H.; Passos T. S. Nanoencapsulation improved water solubility and color stability of carotenoids extracted from Cantaloupe melon (Cucumis melo L.). Food Chem. 2019, 270, 562–572. 10.1016/j.foodchem.2018.07.099. 30174087

[ref56] Castro G. M. M. A.; Passos T. S.; Nascimento S. S. D. C.; Medeiros I.; Araújo N. K.; Maciel B. L. L.; Padilha C. E.; Ramalho A. M. Z.; Sousa Júnior F. C.; de Assis C. F. Gelatin nanoparticles enable water dispersibility and potentialize the antimicrobial activity of Buriti (Mauritia flexuosa) oil. BMC Biotechnol. 2020, 20, 55 10.1186/s12896-020-00649-4. 33066751 PMC7566068

[ref57] Xue J.; Davidson P. M.; Zhong Q. Inhibition of Escherichia coli O157:H7 and Listeria monocytognes growth in milk and cantaloupe juice by thymol nanoemulsions prepared with gelatin and lecithin. Food Control 2017, 73, 1499–1506. 10.1016/j.foodcont.2016.11.015.

[ref58] Qiu L.; Zhang M.; Chitrakar B.; Adhikari B.; Yang C. Effects of nanoemulsion-based chicken bone gelatin-chitosan coatings with cinnamon essential oil and rosemary extract on the storage quality of ready-to-eat chicken patties. Food Packag. Shelf Life 2022, 34, 100933 10.1016/j.fpsl.2022.100933.

[ref59] Ghasemi S.; Jafari S. M.; Assadpour E.; Khomeiri M. Nanoencapsulation of d-limonene within nanocarriers produced by pectin-whey protein complexes. Food Hydrocolloids 2018, 77, 152–162. 10.1016/j.foodhyd.2017.09.030.

[ref60] Zand-Rajabi H.; Madadlou A. Citric acid cross-linking of heat-set whey protein hydrogel influences its textural attributes and caffeine uptake and release behaviour. Int. Dairy J. 2016, 61, 142–147. 10.1016/j.idairyj.2016.05.008.

[ref61] Onsekizoglu Bagci P.; Gunasekaran S. Iron-encapsulated cold-set whey protein isolate gel powder - Part 1: Optimisation of preparation conditions andin vitroevaluation. Int. J. Dairy Technol. 2017, 70, 127–136. 10.1111/1471-0307.12317.

[ref62] Pérez-Masiá R.; López-Nicolás R.; Periago M. J.; Ros G.; Lagaron J. M.; López-Rubio A. Encapsulation of folic acid in food hydrocolloids through nanospray drying and electrospraying for nutraceutical applications. Food Chem. 2015, 168, 124–133. 10.1016/j.foodchem.2014.07.051. 25172691

[ref63] Guo Y.; Harris P.; Kaur A.; Pastrana L.; Jauregi P. Characterisation of β-lactoglobulin nanoparticles and their binding to caffeine. Food Hydrocolloids 2017, 71, 85–93. 10.1016/j.foodhyd.2017.04.027.

[ref64] Remondetto G. E.; Paquin P.; Subirade M. Cold Gelation of β-lactoglobulin in the Presence of Iron. J. Food Sci. 2002, 67, 586–595. 10.1111/j.1365-2621.2002.tb10643.x.

[ref65] Zvereva E. L.; Toivonen E.; Kozlov M. V. Changes in species richness of vascular plants under the impact of air pollution: A global perspective. Glob. Ecol. Biogeogr. 2008, 17, 305–319. 10.1111/j.1466-8238.2007.00366.x.

[ref66] Gilbert N. One-third of our greenhouse gas emissions come from agriculture. Nature 2012, 10.1038/nature.2012.11708.

[ref67] Ölmez H.; Kretzschmar U. Potential alternative disinfection methods for organic fresh-cut industry for minimizing water consumption and environmental impact. LWT--Food Sci. Technol. 2009, 42, 686–693. 10.1016/j.lwt.2008.08.001.

[ref68] USDA-ERS. 2019. Irrigation & water use; Rep., US Dep. Agric. Econ. Res. Serv., Washington, DC. https://www.ers.usda.gov/topics/farm-practices-management/irrigation-water-use/*(Accessed Dec 2022)*

[ref69] US Energy Inf. Adm. 2019. Use of energy explained: energy use in industry; Rep., US Energy Inf. Adm.,Washington, DC. https://www.eia.gov/energyexplained/use-of-energy/industry.php *(Accessed Dec 2022)*

[ref70] P.T., Yih; Oria M.; Nesheim M.C. Natl. Res. Counc., Inst. Med., Board Agric. Nat. Resourc. 2015. A Framework for Assessing Effects of the Food System; Natl. Acad. Press: Washington, DC 26203480

[ref71] Chung M. M. S.; Bao Y.; Zhang B. Y.; Le T. M.; Huang J.-Y. Life cycle assessment on environmental sustainability of food processing. Annu. Rev. Food Sci. Technol. 2022, 13, 217–237. 10.1146/annurev-food-062420-014630. 34936816

[ref72] Chapa J.; Salazar Tijerino M. B.; Kipp S.; Cai H.; Huang J.-Y. A comparative life cycle assessment of fresh imported and frozen domestic organic blueberries consumed in Indiana. J. Cleaner Prod. 2019, 217, 716–723. 10.1016/j.jclepro.2019.01.237.

[ref73] Vauchel P.; Colli C.; Pradal D.; Philippot M.; Decossin S.; Dhulster P.; Dimitrov K. Comparative LCA of ultrasound-assisted extraction of polyphenols from chicory grounds under different operational conditions. J. Cleaner Prod. 2018, 196, 1116–1123. 10.1016/j.jclepro.2018.06.042.

[ref74] de Marco I.; Miranda S.; Riemma S.; Iannone R. Environmental assessment of drying methods for the production of apple powders. Int. J. Life Cycle Assess. 2015, 20, 1659–1672. 10.1007/s11367-015-0971-y.

[ref75] Prosapio V.; Norton I.; de Marco I. Optimization of freeze-drying using a life cycle assessment approach: Strawberries’ case study. J. Cleaner Prod. 2017, 168, 1171–1179. 10.1016/j.jclepro.2017.09.125.

[ref76] de Marco I.; Iannone R. Production, packaging and preservation of semi-finished apricots: A comparative life cycle assessment study. J. Food Eng. 2017, 206, 106–117. 10.1016/j.jfoodeng.2017.03.009.

[ref77] Ramírez C. A.; Patel M.; Blok K. From fluid milk to milk powder: Energy use and energy efficiency in the European dairy industry. Energy 2006, 31, 1984–2004. 10.1016/j.energy.2005.10.014.

[ref78] Jungbluth N.; Keller R.; Meili C. Life cycle assessment of a detailed dairy processing model and recommendations for the allocation to single products. Int. J. Life Cycle Assess. 2018, 23, 1806–1813. 10.1007/s11367-017-1392-x.

[ref79] Finnegan W.; Goggins J.; Clifford E.; Zhan X. Global warming potential associated with dairy products in the Republic of Ireland. J. Cleaner Prod. 2017, 163, 262–273. 10.1016/j.jclepro.2015.08.025.

[ref80] Djekic I.; Miocinovic J.; Tomasevic I.; Smigic N.; Tomic N. Environmental life-cycle assessment of various dairy products. J. Cleaner Prod. 2014, 68, 64–72. 10.1016/j.jclepro.2013.12.054.

[ref81] González-García S.; Castanheira É. G.; Dias A. C.; Arroja L. Environmental life cycle assessment of a dairy product: The yoghurt. Int. J. Life Cycle Assess. 2013, 18, 796–811. 10.1007/s11367-012-0522-8.

[ref82] Yan M.; Holden N. M. Life cycle assessment of multi-product dairy processing using Irish butter and milk powders as an example. J. Cleaner Prod. 2018, 198, 215–230. 10.1016/j.jclepro.2018.07.006.

[ref83] González-García S.; Hospido A.; Moreira M. T.; Feijoo G.; Arroja L. Environmental life cycle assessment of a Galician cheese: San Simon da Costa. J. Cleaner Prod. 2013, 52, 253–262. 10.1016/j.jclepro.2013.03.006.

[ref84] L.M.I., Canals; Muñoz I.; Hospido A.; Plassmann K.; McLaren S. 2008. Life cycle assessment (LCA) of domestic *versus* imported vegetables. Case studies on broccoli, salad crops and green beans. CESWork. Pap. 01/08; University of Surrey

[ref85] Ghorani B.; Tucker N. Fundamentals of electrospinning as a novel delivery vehicle for bioactive compounds in food nanotechnology. Food Hydrocolloids 2015, 51, 227–240. 10.1016/j.foodhyd.2015.05.024.

[ref86] Faridi Esfanjani A.; Jafari S. M. Biopolymer nano-particles and natural nano-carriers for nano-encapsulation of phenolic compounds. Colloids Surf., B 2016, 146, 532–543. 10.1016/j.colsurfb.2016.06.053. 27419648

[ref87] Khorshidi S.; Solouk A.; Mirzadeh H.; Mazinani S.; Lagaron J. M.; Sharifi S.; Ramakrishna S. A review of key challenges of electrospun scaffolds for tissue-engineering applications. J. Tiss. Eng. Regen. Med. 2016, 10, 715–738. 10.1002/term.1978. 25619820

[ref88] López-Rubio A.; Sanchez E.; Sanz Y.; Lagaron J. M. Encapsulation of living bifidobacterial in ultrathin PVOH electrospun fibers. Biomacromolecules 2009, 10, 2823–2829. 10.1021/bm900660b. 19817490

[ref89] Loscertales I. G.; Barrero A.; Guerrero I.; Cortijo R.; Marquez M.; GAÑÁN-CALVO A. M. Micro/nano encapsulation *via* electrified co-axial liquid jets. Science 2002, 295, 1695–1698. 10.1126/science.1067595. 11872835

[ref90] F., Li; Song Y.; Zhao Y. 2010. Core-Shell Nanofibers: Nano Channel and Capsule by Coaxial Electrospinning; INTECH Conference 419–438, 10.5772/8166.

[ref91] Kayaci F.; Uyar T. Encapsulation of vanillin/cyclodextrin inclusion complex in electrospun polyvinyl alcohol (PVA) nanowebs: Prolonged shelf-life and high temperature stability of vanillin. Food Chem. 2012, 133, 641–649. 10.1016/j.foodchem.2012.01.040.

[ref92] Kruncinska I.; Komisarczyk A.; Chrzanowski M.; Gliścińska E.; Wrzosek H. Electrostatic field in electrospinning with a multicapillary head—modelling and experiment. Fibers Text. East. Eur. 2009, 74, 38–44.

[ref93] Petrik S.; Maly M. Production spinneret-less electrospinning nanofiber technology. MRS Online Proc. Libr. 2010, 1240, 307 10.1557/PROC-1240-WW03-07.

[ref94] https://www.nanolayr.com/most-innovative-deep-tech-company-in-new-zealand-nanolayr/ (Accessed Jan 2023)

[ref95] Hao S.; Wang Y.; Wang B.; Deng J.; Liu X.; Liu J. Rapid preparation of pH-sensitive polymeric nanoparticle with high loading capacity using electrospray for oral drug delivery. Mater. Sci. Eng., C 2013, 33, 4562–4567. 10.1016/j.msec.2013.07.009. 24094160

[ref96] Bock N.; Woodruff M. A.; Hutmacher D. W.; Dargaville T. R. Electrospraying, a reproducible method for production of polymeric microspheres for biomedical applications. Polymer 2011, 3, 131–149. 10.3390/polym3010131.

[ref97] Ebrahimgol F.; Tavanai H.; Alihosseini F.; Khayamian T. Electrosprayed recovered wool keratin nanoparticles. Polym. Adv. Technol. 2014, 25, 1001–1007. 10.1002/pat.3342.

[ref98] Fabra M. J.; López-Rubio A.; Lagaron J. M. Use of the electrohydrodynamic process to develop active/bioactive bilayer films for food packaging applications. Food Hydrocolloids 2016, 55, 11–18. 10.1016/j.foodhyd.2015.10.026.

[ref99] Gómez-Estaca J.; Gavara R.; Hernández-Muñoz P. Encapsulation of curcumin in electrosprayed gelatin microspheres enhances its bioaccessibility and widens its uses in food applications. Innov. Food Sci. Emerg. Technol. 2015, 29, 302–307. 10.1016/j.ifset.2015.03.004.

[ref100] Moghaddam M. K.; Mortazavi S. M.; Khayamian T. Preparation of calcium alginate microcapsules containing n-nonadecane by a melt coaxial electrospray method. J. Electrostat. 2015, 73, 56–64. 10.1016/j.elstat.2014.10.013.

[ref101] Zhang L.; Huang J.; Si T.; Xu R. X. Coaxial electrospray of microparticles and nanoparticles for biomedical applications. Expert Rev. Med. Devices 2012, 9, 595–612. 10.1586/erd.12.58. 23249155 PMC3618984

[ref102] Pérez-Masiá R.; Lagaron J. M.; Lopez-Rubio A. Morphology and Stability of Edible Lycopene-Containing Micro- and Nanocapsules Produced Through Electrospraying and Spray Drying. Food Bioprocess Technol. 2015, 8, 459–470. 10.1007/s11947-014-1422-7.

[ref103] Kumar C.; Karim M. A.; Joardder M. U. H. Intermittent drying of food products: A critical review. J. Food Eng. 2014, 121, 48–57. 10.1016/j.jfoodeng.2013.08.014.

[ref104] Darvishi H.; Azadbakht M.; Rezaeiasl A.; Farhang A. Drying characteristics of sardine fish dried with microwave heating. J. Saudi Soc. Agric. Sci. 2013, 12, 121–127. 10.1016/j.jssas.2012.09.002.

[ref105] Pereira N. R.; Marsaioli A. Jr.; Ahrné L. M. Effect of microwave power, air velocity and temperature on the final drying of osmotically dehydrated bananas. J. Food Eng. 2007, 81, 79–87. 10.1016/j.jfoodeng.2006.09.025.

[ref106] Xie X.; Li X.; Zhang C.; Jia W.; Li Y.; Sun H.; Mu G. Combined mid-infrared and hot air drying reduces energy-consumption and improves quality of jerky. Trans. Chin. Soc. Agric. Eng. 2013, 29, 217–226. 10.3969/j.issn.1002-6819.2013.23.030.

[ref107] Zhang J.; Zhang M.; Shan L.; Fang Z. Microwave-vacuum heating parameters for processing savory crisp bighead carp (*Hypophthalmichthys nobilis*) slices. J. Food Eng. 2007, 79, 885–891. 10.1016/j.jfoodeng.2006.03.008.

[ref108] Botha G. E.; Oliveira J. C.; Ahrné L. Quality optimisation of combined osmotic dehydration and microwave assisted air drying of pineapple using constant power emission. Food Bioprod. Process. 2012, 90, 171–179. 10.1016/j.fbp.2011.02.006.

[ref109] Wojdyło A.; Figiel A.; Lech K.; Nowicka P.; Oszmiański J. Effect of convective and vacuum–microwave drying on the bioactive compounds, color, and antioxidant capacity of sour cherries. Food Bioprocess Technol. 2014, 7, 829–841. 10.1007/s11947-013-1130-8.

[ref110] Mee-Ngern B.; Lee S. J.; Choachamnan J.; Boonsupthip W. Penetration of juice into rice through vacuum drying. LWT--Food Sci. Technol. 2014, 57, 640–647. 10.1016/j.lwt.2014.02.001.

[ref111] Ozkahraman B. C.; Sumnu G.; Sahin S. Effect of different flours on quality of legume cakes to be baked in microwave-infrared combination oven and conventional oven. J. Food Sci. Technol. 2016, 53, 1567–1575. 10.1007/s13197-015-2101-z. 27570282 PMC4984705

[ref112] Askari G. R.; Emam-Djomeh Z.; Mousavi S. M. Heat and mass transfer in apple cubes in a microwave-assisted fluidized bed drier. Food Bioprod. Process. 2013, 91, 207–215. 10.1016/j.fbp.2012.09.007.

[ref113] Wang D.; Zhang M.; Wang Y.; Martynenko A. Effect of pulsed-spouted bed microwave freeze drying on quality of apple cuboids. Food Bioprocess Technol. 2018, 11, 941–952. 10.1007/s11947-018-2061-1.

[ref114] Liu Y.; Sun Y.; Yu H. Y.; Yin X.; Li X.; Duan X. Hot air drying of purple-fleshed sweet potato with contact ultrasound assistance. Drying Technol. 2016, 35, 564–576. 10.1080/07373937.2016.1193867.

[ref115] Speckhahn A.; Srzednicki G.; Desai D. K. Drying of beef in superheated steam. Drying Technol. 2010, 28, 1072–1082. 10.1080/07373937.2010.505547.

[ref116] Rao L.; Feeherry F. E.; Ghosh S.; Liao X.; Lin X.; Zhang P.; Li Y.; Doona C. J.; Setlow P. Effects of lowering water activity by various humectants on germination of spores of Bacillus species with different germinants. Food Microbiol. 2018, 72, 112–127. 10.1016/j.fm.2017.11.012. 29407388

[ref117] Torti M. J.; Sims C. A.; Adams C. M.; Sarnoski P. J. Polysaccharides as alternative moisture retention agents for shrimp. J. Food Sci. 2016, 81, S728–S735. 10.1111/1750-3841.13242. 26849189

[ref118] V., Rupasinghe; Handunkutti P.; Joshi A.P.K.; Pitts N.L. 2010. Non-fried apple food products and processes for their preparation. US Patent US20100159082.

[ref119] Thippeswamy L.; Venkateshaiah B. V.; Patil S. B. Effect of modified atmospheric packaging on the shelf stability of paneer prepared by adopting hurdle technology. J. Food Sci. Technol. 2011, 48, 230–235. 10.1007/s13197-010-0155-5. 23572739 PMC3551065

[ref120] Kunová S.; Čuboň J.; Bučko O.; Kačániová M.; Tkáčová J.; Hleba L.; Haščík P.; Lopašovský L. Evaluation of dried salted pork ham and neck quality. Potravinarstvo 2015, 9, 509–514. 10.5219/530.

[ref121] Li X.; Xie X.; Zhang C.-h.; Zhen S.; Jia S. Role of mid- and far-infrared for improving dehydration efficiency in beef jerky drying. Drying Technol. 2018, 36, 283–293. 10.1080/07373937.2017.1326129.

[ref122] Ling J.; Teng Z. S.; Lin H. J.; Wen H. Infrared drying kinetics and moisture diffusivity modeling of pork. Int. J. Agric. Biol. Eng. 2017, 10, 302–311. 10.3965/j.ijabe.20171003.2518.

[ref123] Chandrasekaran S.; Ramanathan S.; Basak T. Microwave food processing—A review. Food Res. Int. 2013, 52, 243–261. 10.1016/j.foodres.2013.02.033.

[ref124] John D.; Ramaswamy H. S. Pulsed light technology to enhance food safety and quality: A mini-review. Curr. Opin. Food Sci 2018, 23, 70–79. 10.1016/j.cofs.2018.06.004.

[ref125] Ehlermann D. A. E. The early history of food irradiation. Radiat. Phys. Chem. 2016, 129, 10–12. 10.1016/j.radphyschem.2016.07.024.

[ref126] M.N.C., Harder; Arthur V.; Arthur P. B. 2016. Irradiation of foods: Processing technology and effects on nutrients: Effect of ionizing radiation on food components. In: Encyclopedia of Food and Health; B., Caballero; Finglas P.M.; Toldra F. F. (Eds.), 476–81. Academic Press: Oxford.

[ref127] Jumah R.; Al-Asheh S.; Banat F.; Al-Zoubi K. Electroosmotic dewatering of tomato paste suspension under an electric field. Drying Technol. 2005, 23, 1465–1475. 10.1081/DRT-200063524.

[ref128] Chen H.; Mujumdar A. S.; Ragbaran G. S. V. Laboratory experiments on electroosmotic dewatering of vegetable sludge and mine tailings. Drying Technol. 1996, 14, 2435–2445. 10.1080/07373939608917215.

[ref129] de Marco I.; Riemma S.; Iannone R. Environmental analysis of a mashed tomato production: an Italian case study. Chem. Eng. Trans. 2017, 57, 1825–1830. 10.3303/CET1757305.

[ref130] S.M., Jafari 2017. Nanoencapsulation Technologies for the Food and Nutraceutical Industries; ISBN: 9780128113646–0128113642. Academic Press.

[ref131] Gharsallaoui A.; Roudaut G.; Chambin O.; Voilley A.; Saurel R. Applications of spray-drying in microencapsulation of food ingredients: an overview. Food Res. Int. 2007, 40, 1107–1121. 10.1016/j.foodres.2007.07.004.

[ref132] Arpagaus C. Nano Spray Dryer B-90: Literature review and applications. Büchi Inf Bull 2011, 63 ().

[ref133] https://assets.buchi.com/image/upload/v1629464619/pdf/Brochures/SB_11592841_Spray_Drying_Encapsulation_es.pdf (Accessed Jun 2023)

[ref134] Aganovic K.; Smetana S.; Grauwet T.; Toepfl S.; Mathys A.; van Loey A.; Heinz V. Pilot scale thermal and alternative pasteurization of tomato and watermelon juice: An energy comparison and life cycle assessment. J. Cleaner Prod. 2017, 141, 514–525. 10.1016/j.jclepro.2016.09.015.

[ref135] Fantin V.; Buttol P.; Pergreffi R.; Masoni P. Life cycle assessment of Italian high quality milk production: A comparison with an EPD study. J. Cleaner Prod. 2012, 28, 150–159. 10.1016/j.jclepro.2011.10.017.

[ref136] G., Doublet; Jungbluth N.; Schori M.; Salome S. 2013. Life cycle assessment of orange juice. SENSE Proj. 288974, 7th Framework Progr; Eur. Comm.: Brussels, Belg.

[ref137] Khanali M.; Kokei D.; Aghbashlo M.; Nasab F. K.; Hosseinzadeh-Bandbafha H.; Tabatabaei M. Energy flow modeling and life cycle assessment of apple juice production: Recommendations for renewable energies implementation and climate change mitigation. J. Cleaner Prod. 2020, 246, 118997 10.1016/j.jclepro.2019.118997.

[ref138] Kim D.; Thoma G.; Nutter D.; Milani F.; Ulrich R.; Norris G. Life cycle assessment of cheese and whey production in the USA. Int. J. Life Cycle Assess. 2013, 18, 1019–1035. 10.1007/s11367-013-0553-9.

[ref139] González-García S.; Castanheira É. G.; Dias A. C.; Arroja L. Environmental performance of a Portuguese mature cheese-making dairy mill. J. Cleaner Prod. 2013, 41, 65–73. 10.1016/j.jclepro.2012.10.010.

[ref140] Madoumier M.; Trystram G.; Sebastian P.; Collignan A. Towards a holistic approach for multi-objective optimization of food processes: A critical review. Trends Food Sci. Technol. 2019, 86, 1–15. 10.1016/j.tifs.2019.02.002.

[ref141] J., Burri; Bertoli C.; Stadler R.H. 2009. Chapter 9: Food processing and nutritional aspects. In Process-induced food toxicants; ed. Stadler R. H.; Lineback D. R.; Hoboken N. J.: Wiley.

[ref142] Taylor M. H.; Tsai H.-C.; Rasco B.; Tang J.; Zhu M.-J. Stability of *Listeria monocytogenes* in wheat flour during extended storage and isothermal treatment. Food Control 2018, 91, 434–439. 10.1016/j.foodcont.2018.04.008.

[ref143] Lespinard A. R.; Bambicha R. R.; Mascheroni R. H. Quality parameters assessment in kiwi jam during pasteurization. Modelling and optimization of the thermal process. Food Bioprod. Process 2012, 90, 799–808. 10.1016/j.fbp.2012.03.001.

[ref144] Sanguinetti A. M.; Del Caro A.; Scanu A.; Fadda C.; Milella G.; Catzeddu P.; Piga A. Extending the shelf life of gluten-free fresh filled pasta by modified atmosphere packaging. LWT--Food Sci. Technol. 2016, 71, 96–101. 10.1016/j.lwt.2016.03.010.

[ref145] Selby T. L.; Berzins A.; Gerrard D. E.; Corvalan C. M.; Grant A. L.; Linton R. H. Microbial heat resistance of *Listeria monocytogenes* and the impact on ready-to-eat meat quality after post-package pasteurization. Meat Sci. 2006, 74, 425–434. 10.1016/j.meatsci.2006.02.018. 22063046

[ref146] Ribeiro G. P.; Villas-Bôas J. K.; Spinosa W. A.; Prudencio S. H. Influence of freezing, pasteurization and maturation on Tiúba honey quality. LWT--Food Sci. Technol. 2018, 90, 607–612. 10.1016/j.lwt.2017.12.072.

[ref147] H., Deeth 2010. Improving UHT processing and UHT milk products, in: Improving the safety and quality of milk; Woodhead Publishing: Woodhead Publishing Series in Food Science, Technology and Nutrition, Mansel W.; Griffiths Eds., pp. 302–329, 10.1533/9781845699420.4.302.

[ref148] M.N., Ramesh 2003. Sterilization of foods, in Encyclopedia of Food Sciences and Nutrition (Second Edition); Academic Press.

[ref149] Pataro G.; Barca G. M. J.; Pereira R. N.; Vicente A. A.; Teixeira J. A.; Ferrari G. Quantification of metal release from stainless steel electrodes during conventional and pulsed ohmic heating. Innovative Food Sci. Emerg. Technol. 2014, 21, 66–73. 10.1016/j.ifset.2013.11.009.

[ref150] Cappato L. P.; Ferreira M. V. S.; Guimaraes J. T.; Portela J. B.; Costa A. L. R.; Freitas M. Q.; Cruz A. G. Ohmic heating in dairy processing: Relevant aspects for safety and quality. Trends Food Sci. Technol. 2017, 62, 104–112. 10.1016/j.tifs.2017.01.010.

[ref151] Pereira R. N.; Rodrigues R. M.; Ramos O. L.; Pinheiro A. L.; Martins J. T.; Teixeira J. A.; Vicente A. A. Electric field processing: Novel perspectives on allergenicity of milk proteins. J. Agric. Food Chem. 2018, 66, 11227–11233. 10.1021/acs.jafc.8b03689. 30296069

[ref152] T., Koutchma 2014. Novel Preservation Applications of UV Light, in: Preservation and Shelf Life Extension; Tatiana, Koutchma Ed.,Academic Press, pp. 45–51, 10.1016/B978-0-12-416621-9.00007-8.

[ref153] Sahoo N.; Sahoo R. K.; Biswas N.; Guha A.; Kuotsu K. Recent advancement of gelatin nanoparticles in drug and vaccine delivery. Int. J. Biol. Macromol. 2015, 81, 317–331. 10.1016/j.ijbiomac.2015.08.006. 26277745

[ref154] Ipsen R.; Otte J. Self-assembly of partially hydrolysed α-lactalbumin. Biotechnol. Adv. 2007, 25, 602–605. 10.1016/j.biotechadv.2007.07.006. 17855040

[ref155] Graveland-Bikker J. F.; de Kruif C. G. Unique milk protein based nanotubes: Food and nanotechnology meet. Trends Food Sci. Technol. 2006, 17, 196–203. 10.1016/j.tifs.2005.12.009.

[ref156] Le Corre D.; Angellier-Coussy H. Preparation and application of starch nanoparticles for nanocomposites: A review. React. Funct. Polym. 2014, 85, 97–120. 10.1016/j.reactfunctpolym.2014.09.020.

[ref157] Wais U.; Jackson A. W.; He T.; Zhang H. Nanoformulation and encapsulation approaches for poorly water-soluble drug nanoparticles. Nanoscale 2016, 8, 1746 10.1039/c5nr07161e. 26731460

[ref158] H., Auweter; Bohn H.; Heger R.; Horn D.; Siegel B.; Siemensmeyer K. 2002. US Patent 6,494,924. Precipitated water-insoluble colorants in colloid disperse form.

[ref159] G.G., Liversidge; Cundy K.C.; Bishop J.; Czekai D. 1991. US Patent 5,145,684. Surface modified drug nanoparticles.

[ref160] Merisko-Liversidge E.; Liversidge G. G.; Cooper E. R. Nanosizing: A formulation approach for poorly-water-soluble compounds. Eur. J. Pharm. Sci. 2003, 18, 113–120. 10.1016/S0928-0987(02)00251-8. 12594003

[ref161] Müller R. H.; Gohla S.; Keck C. M. State of the art of nanocrystals—special features, production, nanotoxicology aspects and intracellular delivery. Eur. J. Pharma. Biopharma. 2011, 78, 1–9. 10.1016/j.ejpb.2011.01.007. 21266197

[ref162] J.E., Kipp; Wong J.C.T.; Doty M.J.; Rebbeck C.L. 2003. Microprecipitation method for preparing submicron suspensions. US6607784B2.

[ref163] J., Moeschwitzer 2006. Verfahren zur herstellung ultrafeiner submicron-suspensionen. WO2006094808A2.

[ref164] Keck C. M.; Müller R. H. Drug nanocrystals of poorly soluble drugs produced by high pressure homogenisation. Eur. J. Pharm. Biopharm. 2006, 62, 3–16. 10.1016/j.ejpb.2005.05.009. 16129588

[ref165] R.H., Müller; Möschwitzer J. 2009. Method and device for producing very fine particles and coating such particles. WO2007051520.

[ref166] Ilari A.; Duca D.; Toscano G.; Pedretti E. F. Evaluation of cradle to gate environmental impact of frozen green bean production by means of life cycle assessment. J. Cleaner Prod. 2019, 236, 117638 10.1016/j.jclepro.2019.117638.

[ref167] J., Davis; Moates G.K.; Waldron K.W. 2009. High-pressure processing: A step toward sustainability?; Food Safety Magazine, Oct. 1. https://www.food-safety.com/articles/3878-high-pressure-processing-a-steptoward-sustainability- *(Accessed Dec 2022)*

[ref168] J., Davis; Moates G.; Waldron K. 2010. The environmental impact of pulsed electric field treatment and high pressure processing: The example of carrot juice. In Case Studies in Novel Food Processing Technologies; ed.C, Doona; Kustin K; Feehery F pp. 103–15. Woodhead Publ: Cambridge, UK.

[ref169] Pardo G.; Zufía J. Life cycle assessment of food-preservation technologies. J. Cleaner Prod. 2012, 28, 198–207. 10.1016/j.jclepro.2011.10.016.

[ref170] Valsasina L.; Pizzol M.; Smetana S.; Georget E.; Mathys A.; Heinz V. Life cycle assessment of emerging technologies: The case of milk ultra-high pressure homogenisation. J. Cleaner Prod. 2017, 142, 2209–2217. 10.1016/j.jclepro.2016.11.059.

[ref171] Mikhaylin S.; Patouillard L.; Margni M.; Bazinet L. Milk protein production by a more environmentally sustainable process: bipolar membrane electrodialysis coupled with ultrafiltration. Green Chem. 2018, 20, 449–456. 10.1039/C7GC02154B.

[ref172] P., Zeuthen; Bøgh-Sørensen L. 2003. Food preservation techniques; Woodhead Publishing: Cambridge, UK, 10.1533/9781855737143.

[ref173] Li D.; Zhu Z.; Sun D.-W. Effects of freezing on cell structure of fresh cellular food materials: A review. Trends Food Sci. Technol. 2018, 75, 46–55. 10.1016/j.tifs.2018.02.019.

[ref174] Lowithun N.; Charoenrein S. Influence of osmodehydrofreezing with different sugars on the quality of frozen rambutan. Int. J. Food Sci. Technol. 2009, 44, 2183–2188. 10.1111/j.1365-2621.2009.02058.x.

[ref175] Zhao J.-H.; Hu R.; Xiao H.-W.; Yang Y.; Liu F.; Gan Z.-Y.; Ni Y.-Y. Osmotic dehydration pretreatment for improving the quality attributes of frozen mango: Effects of different osmotic solutes and concentrations on the samples. Int. J. Food Sci. Technol. 2014, 49, 960–968. 10.1111/ijfs.12388.

[ref176] Dermesonlouoglou E. K.; Giannakourou M. C.; Taoukis P. Stability of dehydrofrozen tomatoes pretreated with alternative osmotic solutes. J. Food Eng. 2007, 78, 272–280. 10.1016/j.jfoodeng.2005.09.026.

[ref177] D., Kleppner; Kolenkow R. 2018. An introduction to mechanics; Cambridge University Press: Cambridge, MA.

[ref178] Hygreeva D.; Pandey M. C. Novel approaches in improving the quality and safety aspects of processed meat products through high pressure processing technology - A review. Trends Food Sci. Technol. 2016, 54, 175–185. 10.1016/j.tifs.2016.06.002.

[ref179] Sevenich R.; Rauh C.; Knorr D. A scientific and interdisciplinary approach for high pressure processing as a future toolbox for safe and high quality products: A review. Innovative Food Sci. Emerg. Technol. 2016, 38, 65–75. 10.1016/j.ifset.2016.09.013.

[ref180] Sandoval Murillo J. L.; Osen R.; Hiermaier S.; Ganzenmüller G. Towards understanding the mechanism of fibrous texture formation during high-moisture extrusion of meat substitutes. J. Food Eng. 2019, 242, 8–20. 10.1016/j.jfoodeng.2018.08.009.

[ref181] Koprivica G. B.; Mišljenović N. M.; Lević L. B.; Jevrić L. R.; Filipčev B. V. Osmotic dehydration of carrot in sugar beet molasses: Mass transfer kinetics. Acta Period. Technol. 2010, 41, 1–55. 10.2298/APT1041047K.

[ref182] Cao H.; Zhang M.; Mujumdar A. S.; Du W.-h.; Sun J.-c. Optimization of osmotic dehydration of kiwifruit. Drying Technol. 2006, 24, 89–94. 10.1080/07373930500538741.

[ref183] Viana A. D.; Corrêa J. L. G.; Justus A. Optimisation of the pulsed vacuum osmotic dehydration of cladodes of fodder palm. Int. J. Food Sci. Technol. 2014, 49, 726–732. 10.1111/ijfs.12357.

[ref184] Chwastek A. Methods to increase the rate of mass transfer during osmotic dehydration of foods. Acta Sci. Pol., Technol. Aliment. 2014, 13, 341–350. 10.17306/J.AFS.2014.4.1. 28067476

[ref185] Gong Z.; Gao L.; An J.; Min Z.; Mujumdar A. S.; Sun J. Effects of predrying and vacuum impregnation with nano-calcium carbonate solution on strawberries, carrots, corn, and blueberries. Drying Technol. 2009, 28, 36–41. 10.1080/07373930903423913.

[ref186] Xie J.; Zhao Y. Nutritional enrichment of fresh apple (*Royal Gala*) by vacuum impregnation. Int. J. Food Sci. Nutr. 2003, 54, 387–398. 10.1080/09637480310001595261. 12907403

[ref187] Derossi A.; Iliceto A.; De Pilli T.; Severini C. Application of vacuum impregnation with anti-freezing proteins to improve the quality of truffles. J. Food Sci. Technol. 2015, 52, 7200–7208. 10.1007/s13197-015-1843-y.

[ref188] Barat J. M.; Grau R.; Pagán-Moreno M. J.; Fito P. Replacement of pile salting by simultaneous brine thawing-salting in Spanish cured ham manufacturing. Meat Sci. 2004, 66, 603–608. 10.1016/S0309-1740(03)00176-1. 22060870

[ref189] Lima M. M. D.; Tribuzi G.; Souza J. A. R. D.; Souza I. G. D.; Laurindo J. B.; Carciofi B. A. M. Vacuum impregnation and drying of calcium-fortified pineapple snacks. LWT--Food Sci. Technol. 2016, 72, 501–509. 10.1016/j.lwt.2016.05.016.

[ref190] Zhao Y.; Xie J. Practical applications of vacuum impregnation in fruit and vegetable processing. Trends Food Sci. Technol. 2004, 15, 434–451. 10.1016/j.tifs.2004.01.008.

[ref191] H., Feng; Barbosa-Cánovas G.V.; Weiss J. 2011. Ultrasound technologies for food and bioprocessing. New York: Springer, 10.1007/978-1-4419-7472-3.

[ref192] Simal S.; Benedito J.; Sánchez E. S.; Rosselló C. Use of ultrasound to increase mass transport rates during osmotic dehydration. J. Food Eng. 1998, 36, 323–336. 10.1016/S0260-8774(98)00053-3.

[ref193] Goula A. M.; Kokolaki M.; Daftsiou E. Use of ultrasound for osmotic dehydration. The case of potatoes. Food Bioprod. Process. 2017, 105, 157–170. 10.1016/j.fbp.2017.07.008.

[ref194] Oliveira F. I. P.; Rodrigues S.; Fernandes F. A. N. Production of low calorie Malay apples by dual stage sugar substitution with Stevia-based sweetener. Food Bioprod. Process. 2012, 90, 713–718. 10.1016/j.fbp.2012.02.002.

[ref195] Tadros T.; Izquierdo P.; Esquena J.; Solans C. Formation and stability of nano-emulsions. Adv. Colloid Interface Sci. 2004, 108-109, 303–318. 10.1016/j.cis.2003.10.023. 15072948

[ref196] Walker R.; Decker E. A.; McClements D. J. Development of food-grade nanoemulsions and emulsions for delivery of omega-3 fatty acids: Opportunities and obstacles in the food industry. Food Funct. 2015, 6, 42–54. 10.1039/c4fo00723a. 25384961

[ref197] S.A., Chime; Kenechukwu F.C.; Attama A.A. 2014. Nanoemulsions—advances in formulation, characterization and applications in drug delivery. In: Ali D.S. (Ed.), Application of Nanotechnology in Drug Delivery; In Tech: Croatia, pp. 77–111.

[ref198] Schubert H.; Ax K.; Behrend O. Product engineering of dispersed systems. Trends Food Sci. Technol. 2003, 14, 9–16. 10.1016/S0924-2244(02)00245-5.

[ref199] Floury J.; Desrumaux A.; Lardieres J. Effect of high-pressure homogenization on droplet size distributions and rheological properties of model oil-in-water emulsions. Innov. Food Sci. Emerg. Technol. 2000, 1, 127–134. 10.1016/S1466-8564(00)00012-6.

[ref200] D.J., McClements 2015. Food Emulsions: Principles, Practices, and Techniques; third ed. CRC Press, Boca Raton.

[ref201] Radtke M.; Müller R. H. Nanostructured lipid drug carriers. New Drugs 2001, 2, 48–52. 10.1201/b18868.

[ref202] Lee L.; Hancocks R.; Noble I.; Norton I. T. Production of water-in-oil nanoemulsions using high pressure homogenisation: a study on droplet break-up. J. Food Eng. 2014, 131, 33–37. 10.1016/j.jfoodeng.2014.01.024.

[ref203] Henry J. V. L.; Fryer P. J.; Frith W. J.; Norton I. T. The influence of phospholipids and food proteins on the size and stability of model sub-micron emulsions. Food Hydrocolloids 2010, 24, 66–71. 10.1016/j.foodhyd.2009.08.006.

[ref204] Donsì F.; Annunziata M.; Vincensi M.; Ferrari G. Design of nanoemulsion-based delivery systems of natural antimicrobials: effect of the emulsifier. J. Biotechnol. 2012, 159, 342–350. 10.1016/j.jbiotec.2011.07.001. 21763730

[ref205] S.M., Jafari; Fathi M.; Mandala I. 2015. Emerging product formation. In: Food Waste Recovery: Processing Technologies and Industrial Techniques; Elsevier Inc.: pp. 293–317.

[ref206] Chemat F.; Zill-e-Huma M. K. K.; Khan M. K. Applications of ultrasound in food technology: processing, preservation and extraction. Ultrason. Sonochem. 2011, 18, 813–835. 10.1016/j.ultsonch.2010.11.023. 21216174

[ref207] Freitas S.; Hielscher G.; Merkle H. P.; Gander B. Continuous contact- and contamination-free ultrasonic emulsification—A useful tool for pharmaceutical development and production. Ultrason. Sonochem. 2006, 13, 76–85. 10.1016/j.ultsonch.2004.10.004. 16223691

[ref208] Pascual-Pineda L. A.; Flores-Andrade E.; Jiménez-Fernández M.; Beristain C. I. Kinetic and thermodynamic stability of paprika nanoemulsions. Int. J. Food Sci. Technol. 2015, 50, 1174–1181. 10.1111/ijfs.12750.

[ref209] Anton N.; Mojzisova H.; Porcher E.; Benoit J. P.; Saulnier P. Reverse micelle-loaded lipid nano-emulsions: new technology for nano-encapsulation of hydrophilic materials. Int. J. Pharm. 2010, 398, 204–209. 10.1016/j.ijpharm.2010.07.039. 20674723

[ref210] Pan K.; Zhong Q. Organic Nanoparticles in Foods: Fabrication, characterization, and utilization. Annu. Rev. Food Sci. Technol. 2016, 7, 245–266. 10.1146/annurev-food-041715-033215. 26735797

[ref211] T.V., Candéa 2013. Study of membrane emulsification process as a pre-step for the microencapsulation of lipid compounds by spray dryind. MSc Thesis. Universidade Nova de Lisboa.

[ref212] Huang Z.; Li X.; Zhang T.; Song Y.; She Z.; Li J.; Deng Y. Progress involving new techniques for liposome preparation. Asian J. Pharm. Sci. 2014, 9, 176–182. 10.1016/j.ajps.2014.06.001.

[ref213] Liu W.; Ye A.; Liu W.; Liu C.; Han J.; Singh H. Behaviour of liposomes loaded with bovine serum albumin during in vitro digestion. Food Chem. 2015, 175, 16–24. 10.1016/j.foodchem.2014.11.108. 25577045

[ref214] Lesoin L.; Crampon C.; Boutin O.; Badens E. Preparation of liposomes using the supercritical anti-solvent (SAS) process and comparison with a conventional method. J. Supercrit. Fluids 2011, 57, 162–174. 10.1016/j.supflu.2011.01.006.

[ref215] Otake K.; Imura T.; Sakai H.; Abe M. Development of a new preparation method of liposomes using supercritical carbon dioxide. Langmuir 2001, 17, 3898–3901. 10.1021/la010122k.

[ref216] Massing U.; Cicko S.; Ziroli V. Dual asymmetric centrifugation (DAC) - A new technique for liposome preparation. J. Controlled Release 2008, 125, 16–24. 10.1016/j.jconrel.2007.09.010. 18023907

[ref217] Pham T. T.; Jaafar-Maalej C.; Charcosset C.; Fessi H. Liposome and niosome preparation using a membrane contactor for scale-up. Colloids Surf., B 2012, 94, 15–21. 10.1016/j.colsurfb.2011.12.036. 22326648

[ref218] Laouini A.; Fessi H.; Charcosset C. Membrane emulsification: A promising alternative for vitamin E encapsulation within nano-emulsion. J. Membr. Sci. 2012, 423-424, 85–96. 10.1016/j.memsci.2012.07.031.

[ref219] Wang T.; Deng Y.; Geng Y.; Gao Z.; Zou J.; Wang Z. Preparation of submicron unilamellar liposomes by freeze-drying double emulsions. Biochim. Biophys. Acta, Biomembr. 2006, 1758, 222–231. 10.1016/j.bbamem.2006.01.023. 16563340

[ref220] Chen C.; Han D.; Cai C.; Tang X. An overview of liposome lyophilization and its future potential. J. Controlled Release 2010, 142, 299–311. 10.1016/j.jconrel.2009.10.024. 19874861

[ref221] Escribà-Gelonch M.; Bricout J.; Hessel V. Circular economy metrics for the photo-high-p,T continuous multistep synthesis of vitamin D_3_. ACS Sustainable Chem. Eng. 2021, 9, 1867–1879. 10.1021/acssuschemeng.0c08330.

[ref222] Escribà M.; Eras J.; Villorbina G.; Balcells M.; Blanch C.; Barniol N.; Canela R. Use of crude glycerol from biodiesel producers and fatty materials to prepare allyl esters. Waste Biomass Valoriz. 2011, 2, 285–290. 10.1007/s12649-011-9073-7.

[ref223] D.I., Santos; Saraiva J.M.A.; Vicente A.A.; Moldao-Martins M. 2019. Methods for determining bioavailability and bioaccessibility of bioactive compounds and nutrients, in: Innovative Thermal and Non-Thermal Processing, Bioaccessibility and Bioavailability of Nutrients and Bioactive Compounds; Elsevier, pp. 23–54, 10.1016/B978-0-12-814174-8.00002-0.

[ref224] Sirohi R.; Pandey J. P.; Gaur V. K.; Gnansounou E.; Sindhu R. Critical overview of biomass feedstocks as sustainable substrates for the production of polyhydroxybutyrate (PHB). Bioresour. Technol. 2020, 311, 123536 10.1016/j.biortech.2020.123536. 32448640

[ref225] Al-Dhabi N. A.; Ponmurugan K.; Jeganathan P. M. Development and validation of ultrasound-assisted solid-liquid extraction of phenolic compounds from waste spent coffee grounds. Ultrason. Sonochem. 2017, 34, 206–213. 10.1016/j.ultsonch.2016.05.005. 27773237

[ref226] Guandalini B. B. V.; Rodrigues N. P.; Marczak L. D. F. Sequential extraction of phenolics and pectin from mango peel assisted by ultrasound. Food Res. Int. 2019, 119, 455–461. 10.1016/j.foodres.2018.12.011. 30884677

[ref227] Hessel V.; Tran N. N.; Asrami M. R.; Tran Q. D.; Long N. V. D.; Escribà-Gelonch M.; Osorio-Tejada J.; Linke S.; Sundmacher K. Sustainability of green solvents – Review and perspective. Green Chem. 2022, 24, 410–437. 10.1039/D1GC03662A.

[ref228] Pal C. B. T.; Jadeja G. C. Microwave-assisted extraction for recovery of polyphenolic antioxidants from ripe mango (*Mangifera indica L.*) peel using lactic acid/sodium acetate deep eutectic mixtures. Food Sci. Technol. Int. 2020, 26, 78–92. 10.1177/1082013219870010. 31466477

[ref229] Plazzotta S.; Ibarz R.; Manzocco L.; Martín-Belloso O. Optimizing the antioxidant biocompound recovery from peach waste extraction assisted by ultrasounds or microwaves. Ultrason. Sonochem. 2020, 63, 104954 10.1016/j.ultsonch.2019.104954. 31945560

[ref230] Escribà M.; Eras J.; Duran M.; Simon S.; Butchosa C.; Villorbina G.; Balcells M.; Canela R. From glycerol to chlorohydrin esters using a solvent-free system. Microwave irradiation versus conventional heating. Tetrahedron 2009, 65, 10370–10376. 10.1016/j.tet.2009.10.048.

[ref231] Eras J.; Escribà M.; Villorbina G.; Oromí-Farrús M.; Balcells M.; Canela R. A tandem Finkelstein-rearrangement–elimination reaction: A straightforward synthetic route to allyl esters. Tetrahedron 2009, 65, 4866–4870. 10.1016/j.tet.2009.04.042.

[ref232] Escribà M.; Barbut M.; Eras J.; Canela R.; Avilla J.; Balcells M. Synthesis of allyl esters of fatty acids and their ovicidal effect on *Cydia pomonella (L.)*. J. Agric. Food Chem. 2009, 57, 4849–4853. 10.1021/jf900097j. 19489625

[ref233] Eide M. H.; Homleid J. P.; Mattsson B. Life cycle assessment (LCA) of cleaning-in-place processes in dairies. LWT--Food Sci. Technol. 2003, 36, 303–314. 10.1016/S0023-6438(02)00211-6.

[ref234] Segat A.; Misra N. N.; Cullen P. J.; Innocente N. Effect of atmospheric pressure cold plasma (ACP) on activity and structure of alkaline phosphatase. Food Bioprod. Process. 2016, 98, 181–188. 10.1016/j.fbp.2016.01.010.

[ref235] Laroussi M. Low temperature plasma-based sterilization: Overview and state-of-the-art. Plasma Processes Polym. 2005, 2, 391–400. 10.1002/ppap.200400078.

[ref236] Kim B.; Yun H.; Jung S.; Jung Y.; Jung H.; Choe W.; Jo C. Effect of atmospheric pressure plasma on inactivation of pathogens inoculated onto bacon using two different gas compositions. Food Microbiol. 2011, 28, 9–13. 10.1016/j.fm.2010.07.022. 21056769

[ref237] Song H. P.; Kim B.; Choe J. H.; Jung S.; Moon S. Y.; Choe W.; Jo C. Evaluation of atmospheric pressure plasma to improve the safety of sliced cheese and ham inoculated by 3-strain cocktail *Listeria monocytogenes*. Food Microbiol. 2009, 26, 432–436. 10.1016/j.fm.2009.02.010. 19376467

[ref238] Yun H. J.; Binna K.; Jung S.; Kruk Z. A.; Danbee K.; Wonho C.; Cheorun J. Inactivation of *Listeria monocytogenes* inoculated on disposable plastic tray, aluminum foil, and paper cup by atmospheric pressure plasma. Food Control 2010, 21, 1182–1186. 10.1016/j.foodcont.2010.02.002.

[ref239] Choi S.; Puligundla P.; Mok C. Impact of corona discharge plasma treatment on microbial load and physicochemical and sensory characteristics of semi-dried squid (*Todarodes pacificus*). Food Sci. Biotechnol. 2017, 26, 1137–1144. 10.1007/s10068-017-0137-8. 30263646 PMC6049553

[ref240] Zhou D.; Li T.; Cong K.; Suo A.; Wu C. Influence of cold plasma on quality attributes and aroma compounds in fresh-cut cantaloupe during low temperature storage. LWT 2022, 154, 112893 10.1016/j.lwt.2021.112893.

